# In Situ Preparation of Crosslinked Polymer Electrolytes for Lithium Ion Batteries: A Comparison of Monomer Systems

**DOI:** 10.3390/polym12081707

**Published:** 2020-07-30

**Authors:** Eike T. Röchow, Matthias Coeler, Doris Pospiech, Oliver Kobsch, Elizaveta Mechtaeva, Roland Vogel, Brigitte Voit, Kristian Nikolowski, Mareike Wolter

**Affiliations:** 1 Leibniz-Institut für Polymerforschung Dresden e.V., Hohe Str. 6, 01069 Dresden, Germany; eike.roechow@web.de (E.T.R.); kobsch@ipfdd.de (O.K.); rolandvogel504@gmail.com (R.V.); voit@ipfdd.de (B.V.); 2 Organic Chemistry of Polymers, Technische Universität Dresden, 01062 Dresden, Germany; 3 Fraunhofer-Institut für Keramische Technologien und Systeme IKTS, Winterbergstr. 28, 01277 Dresden, Germany; matthias.coeler@ikts.fraunhofer.de (M.C.); kristian.nikolowski@ikts.fraunhofer.de (K.N.); mareike.wolter@ikts.fraunhofer.de (M.W.); 4 Department of High-Molecular Compounds Chemistry, St. Petersburg University, Universitetskaya Emb., 7/9, Saint-Petersburg 199034, Russia; mechtaeva.lisa@gmail.com

**Keywords:** lithium ion battery, polymer electrolyte, polymeric ionic liquid, photopolymerization, ionic conductivity, electrochemical stability

## Abstract

Solid polymer electrolytes for bipolar lithium ion batteries requiring electrochemical stability of 4.5 V vs. Li/Li^+^ are presented. Thus, imidazolium-containing poly(ionic liquid) (PIL) networks were prepared by crosslinking UV-photopolymerization in an in situ approach (i.e., to allow preparation directly on the electrodes used). The crosslinks in the network improve the mechanical stability of the samples, as indicated by the free-standing nature of the materials and temperature-dependent rheology measurements. The averaged mesh size calculated from rheologoical measurements varied between 1.66 nm with 10 mol% crosslinker and 4.35 nm without crosslinker. The chemical structure of the ionic liquid (IL) monomers in the network was varied to achieve the highest possible ionic conductivity. The systematic variation in three series with a number of new IL monomers offers a direct comparison of samples obtained under comparable conditions. The ionic conductivity of generation II and III PIL networks was improved by three orders of magnitude, to the range of 7.1 × 10^−6^ S·cm^−1^ at 20 °C and 2.3 × 10^−4^ S·cm^−1^ at 80 °C, compared to known poly(vinylimidazolium·TFSI) materials (generation I). The transition from linear homopolymers to networks reduces the ionic conductivity by about one order of magnitude, but allows free-standing films instead of sticky materials. The PIL networks have a much higher voltage stability than PEO with the same amount and type of conducting salt, lithium bis(trifluoromethane sulfonyl)imide (LiTFSI). GII-PIL networks are electrochemically stable up to a potential of 4.7 V vs. Li/Li^+^, which is crucial for a potential application as a solid electrolyte. Cycling (cyclovoltammetry and lithium plating-stripping) experiments revealed that it is possible to conduct lithium ions through the GII-polymer networks at low currents. We concluded that the synthesized PIL networks represent suitable candidates for solid-state electrolytes in lithium ion batteries or solid-state batteries.

## 1. Introduction

At present, lithium ion batteries are still considered the power source of choice for mobile applications, e.g., in consumer electronics, and for next generation hybrid and electric vehicles due to the mature, highly advanced technology and relatively high energy efficiency [1,2,3,4,5,6]. Automotive applications require large-area batteries. Scaling-up the geometry and chemistry with standard electrochemistry is problematic with respect to battery manufacturing and safety. Despite all efforts to develop and install solid-state electrolytes, the state-of-the-art in lithium ion batteries is still the use of liquid electrolytes (i.e., mixtures of organic solvents with conducting salts [7]). They must penetrate into the pores of the electrodes and a thorough wetting has to be achieved. This time-consuming process is the bottleneck in the production chain. Liquid electrolytes cause major problems in battery safety and thermal management upon leakage. Local overheating and short circuits cause a variety of reactions between the battery components and yield evaporation of the water and oxygen-sensitive electrolyte. These processes cause exothermic reactions that dramatically increase the battery temperature, so generate ignition of the liquid electrolyte and an explosion, which becomes even more dramatic in large-area batteries. Furthermore, liquid electrolytes with standard chemistry and electrochemical stabilities up to 3.5 V vs. Li/Li^+^ cannot be applied in electrochemical systems using high voltage electrodes (e.g., lithium nickel manganese oxide LiNi_0.5_Mn_1.5_O_4_ (LNMO) with an electrochemical stability up to 4.5 V vs. Li/Li^+^). Therefore, the development of safer electrolytes has been intensively pursued following different concepts that are summarized in a number of excellent reviews [3,7,8,9,10,11,12,13,14]. The concepts included (i) replacement of moisture-sensitive salts with less sensitive conducting salts, e.g., salts with non-coordinating anions with extensive charge delocalization, such as lithium bis(trifluoromethane sulfonyl)imide (LiTFSI) [9,15,16]; (ii) substitution of the flammable organic liquids by non-flammable ionic liquids [8,17,18,19,20,21,22] with negligible vapor pressure [23,24]; (iii) incorporation of conducting salts into a swollen polymer (gel electrolytes) [20,25,26,27]; (iv) incorporation of conducting salts into dry polymers to yield solid polymer electrolytes, often reported with poly(ethylene oxide) (PEO) as the matrix to yield solid state electrolytes (SSE) [28,29]; (v) substitution of salts with polymers with ionic sites (often polymeric ionic liquids, PIL) [7,8,12,30,31,32,33,34,35,36,37]; (vi) complete replacement of organics by Garnet-type ceramics [38,39]; and recently, vii) preparation of organic/inorganic hybrids with inorganic nanoparticles such as TiO_2_ to boost ionic conductivity and lithium transference numbers [31,40,41]. The combination of sulfonated polysulfone, Al_2_O_3_ or SiO_2_ nanoparticles, and an IL resulted in polymer electrolytes combining some of those concepts [42,43].

It has to be mentioned that the ionic conductivity of all-solid-state polymer electrolytes is at least two orders of magnitude lower than that of liquid electrolytes. This is usually explained by the different ion conduction mechanisms and types of ion diffusion, as discussed intensively in the literature [44]. In liquid electrolytes, the ions are able to move freely through the solution. Thus, the conductivity in liquids depends mainly on the state of dissociation, ionic properties, and the viscosity of the solution (Nernst–Einstein equation) [44]. In polymer electrolytes, in contrast, the lithium ions are coordinated to parts of the polymer chain, and their diffusivity is strongly coupled to the dynamics of the polymer backbone. The major difference to liquids is that the ions move via hopping from coordination site to coordination site or from cluster to cluster [45,46]. Factors playing a role can be summarized to be (i) segmental dynamics of the polymer; (ii) cation solvation dynamics (of the conducting salt), cation-anion interactions, and (iii) solvation-site connectivity [47,48].

Polymer ionic liquids are the common favorite polymer electrolytes with high potential. The monomers show ionic liquid character at room temperature, while the polymers often do not [49,50]. A variety of basic chemical structures and polymer architectures have been explored in the past to find the most promising structures for high ion conductivity and applications in lithium ion battery cells. One of the most often used basic structures (although many other have been reported [31,32,49,50,51,52]) is the imidazolium ring with the TFSI counter ion, resulting in cationic-type polymers. In these polymers, the basic cationic units can be positioned in the polymer backbone [53], as side chains either in linear [36,37] or in crosslinked polymers [54,55,56,57], together with anions in the side chain (zwitterionic type), in alternating copolymers or blockwise [21,32,46,58] accompanied by anionic side chains [31,54], and in the arms of star-shaped polymers. TFSI, as a counterion for cationic sites, is particularly preferred owing to the high van der Waals volume and week binding affinity to Li^+^ cations [36,46]. Despite all the efforts to develop systems suitable for lithium ion batteries [31], the electrochemical properties of PILs are often not sufficient to ensure proper function in battery cells. Therefore, their applications are still limited and only rare examples are found wherein PILs are applied without additional plasticizers (often low molar mass ionic liquids) [8,31,59]. This is one of the reasons why the research activities are intensively continued in this field.

The study presented here aimed at the development of solid-state polymer electrolytes for application in large-area, bipolar-type lithium ion batteries. These materials have to meet special requirements, in particular, high lithium ion conductivity, electrochemical stability up to 4.5 V vs. Li/Li^+^, high thermal stability, and last but not least, compatibility with the fabrication process for bipolar batteries. The polymer electrolyte should serve both as an ion-conducting binder in composite electrodes, and a separator between the electrodes. In order to achieve these goals, we tried to combine the best of the different concepts reported in the literature. An in situ approach was chosen to adjust the polymer electrolyte material to the processing chain of bipolar batteries. That means that the polymers here were not prepared separately as usually described [31,32,37,60], but directly within the fabrication process of the composite electrodes and the separating layer. To achieve this, the rate of polymerization under the conditions used had to be analyzed, which was done here using Raman spectroscopy.

**Figure 1:**
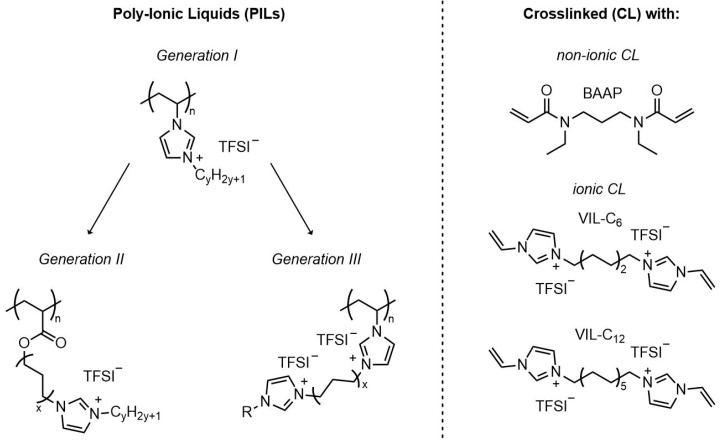
Chemical concept for crosslinked polymer electrolytes based on polymeric ionic liquids studied here (TFSI: bis(trifluoromethanesulfonyl)imide).

The type of materials employed was the group of cationic polymer ionic liquids. From the condensed knowledge reported in the literature, as briefly summarized above, it was assumed that imidazolium-TFSI-based PILs [31,34,35,36,37,53] could serve as suitable candidates and starting points due to sufficient electrochemical stability. For that reason, the incorporation of (ethylene oxide) units was avoided. The most promising chemical concepts described in the literature were combined and further developed here to elaborate suitable materials. The chemical concept to achieve a reliable comparison of systems is illustrated in Figure 1.

Polymerizable imidazolium-based IL monomers with systematically altered chemical structures were synthesized and characterized by NMR spectroscopy to ensure sufficient purity. *Generation I* (GI) (on the left hand side of Figure 1) consisted of vinylimidazolium monomers with N-alkyl substituents with varied alkyl chain length, as described before as linear homopolymers by Delhorbe et al. [37] and others [31,34,35,53,61]. Generation I served as a comparison set. In *generation II* (GII) monomers, an additional alkyl spacer was introduced between acrylate group and the alkylimidazolium group. GII monomers and corresponding linear homopolymers were first reported by Ohno et al. [62,63,64] and Shaplov et al. [65]. A description of properties of the complete series with systematically varied alkyl spacers in particular with respect to electrochemical stability, was not given [62,63]. The number of GII IL monomers was expanded in this study by monomers with longer N-alkyl groups. *Generation III* (GIII) monomers developed here have not been described previously in the literature. They contained two imidazolium groups in order to enhance the number of ionic groups in the monomer which was suggested as one possibility to enhance ionic conductivity [31]. From a structural point of view, they reflect a mixture of the structural features of GI and GII.

These systematic variations should allow the influence of the chemical structure of the monomers on the properties of the resulting polymers, synthesized and analyzed under comparable conditions, to be demonstrated. This is often not the case in the comparison of different studies discussed in the literature. Thus, the influences of impurities, in particular, water traces; sample preparation; measurement setup; and measurement conditions on the ionic conductivity—which is sensitive to all those parameters [66]—can be minimized.

The monomers were employed to prepare homopolymers and crosslinked networks with different crosslinker chemistry and network density. Free-standing PIL films were prepared by mixing the monomers of generation I, II, and III with suitable crosslinkers followed by UV-light initiated free radical polymerization (FRP) as an in situ approach. UV-initiated polymerization to obtain IL-containing networks has been reported before [29,67,68,69,70]. The preparation conditions in this study were kept constant to ensure comparability of the samples according to the information obtained by monitoring the polymerization rate. Information in the course of crosslinking polymerization of PILs can be found only rarely in the literature. The polymerization behavior of the formulations containing monomer, crosslinker, photoinitiator, and conducting salt was examined by Raman microscopy. Two distinct crosslinbkers have been employed: (1) a non-ionic, hydrophilic bifunctional monomer (N,N’-diethyl-1,3-bis(acrylamido)propane, (BAAP)) not utilized so far for polyelectrolyte networks but in dentistry [71,72], and (2) two crosslinkers with two imidazolium groups and different alkyl spacer length were employed (shown on the right hand side of Figure 1). The imidazolium-containing crosslinkers were first reported by von Zamory et al. [33,73]. The incorporation of crosslinkers aimed to ensure a certain mechanical stability of the polyelectrolyte films, which may not be given in materials with very low T_g_.

The free-standing films of networks of all three generations of samples were analyzed thoroughly. The network characteristics were accessed by rheological measurements. They are reported, to the best of our knowledge, for the first time herein, and that offers the possibility to directly correlate ionic conductivity with network density, and to corroborate general assumptions [45] with data. The softening behavior was analyzed by differential scanning calorimetry (DSC) yielding the glass transition temperature (T_g_) of the network serving as a measure for the flexibility of the materials at room temperature. The thermal stability as an important parameter for applications in lithium ion cells was characterized by thermogravimetric analysis (TGA). Electrochemical properties, in particular, the ion conductivity at different temperatures, were measured by electrochemical impedance spectroscopy (EIS). The electrochemical stability was analyzed by linear sweep voltammetry (LSV) and cyclic voltammetry (CV). Lithium plating-stripping experiments (DC) were performed to characterize the lithium ion transport properties.

## 2. Materials and Methods

### 2.1. Materials

Acryloyl chloride (AACl, 96%, Alfa Aesar, Ward Hill, MA, USA), 12-bromo-1-dodecanol (BrC_12_OH, >95%, TCI, Tokyo, Japan), 6-bromo-1-hexanol (BrC_6_OH, >95%, TCI), 9-bromo-1-nonanol (BrC_9_OH, >95%, TCI), 1-butylimidazole (C_4_Im, >98%, TCI), iodobutane (BuI, 99%, Alfa Aesar), 1-ethylimidazole (C_2_Im, >98%, TCI), 1-hexylimidazole (C_6_Im, >98%, IoLiTec Ionic Liquids Technologies GmbH, Heilbronn, Germany), magnesium sulfate (>98%, Sigma Aldrich, Saint louis, MO, USA), 1-methylimidazole (C_1_Im, 99%, Alfa Aesar), methyl tert-butyl ether (MTBE, 99%, Sigma Aldrich), silver nitrate (0.1 M, Sigma Aldrich), triethylamine (TEA, 99%, Alfa Aesar), trimethylbenzoyl diphenylphosphine oxide (TPO, 97%, Sigma Aldrich), and 1-vinylimidazole (VIm, 99%, Sigma Aldrich) were used as received unless otherwise stated. The crosslinker N,N’-diethyl-1,3-bis(acrylamide)-propane, (BAAP), was kindly provided by Ivoclar Vivadent AG (Schaan, FL, USA). Lithium bis(trifluoromethane sulfonyl)imide (LiTFSI, 99%, IoLiTec Ionic Liquids Technologies GmbH, Heilbronn, Germany) was dried under vacuum at 110 °C for 24 h prior to use.

### 2.2. Synthesis of Butyl Vinylimidazolium Iodide

The synthesis of alkyl vinylimidazolium iodides is given as an example for butyl vinylimidazolium iodide (VImC_4_ I) which was synthesized by quaternization of VIm with BuI and subsequent anion exchange. Thus, one equivalent of both VIm and haloalkane (BuI) was dissolved in MTBE. The solution was stirred at 50 °C for 24 h. The resulting monomer was obtained as yellowish, oily liquid and purified by extraction with ethyl acetate. The product was dried in vacuo at 40 °C for several hours. Yield: 73%. ^1^H NMR, ^13^C NMR: see Supporting Information.

### 2.3. Synthesis of Acrylic Imidazolium Bromide ILs

The synthesis of acrylic imidazolium ILs was performed according to the report of Yoshizawa et al. [62,63,64] with small modifications to achieve higher yields. Thus, a mixture of the corresponding bromoalcohol (BrC_6_OH, BrC_9_OH, BrC_12_OH) and 1.2 equivalents of TEA in THF was stirred at room temperature for 1 h. Then, 1.2 equivalents of acryloyl chloride in THF were added slowly under argon atmosphere while cooling to 0 °C. The mixture was stirred for 1 h at 0 °C, followed by heating to 50 °C for 48 h. During this time, the formation of a white precipitate of N(Et)_3_HCl could be observed. The THF was removed and the solid residue was dissolved in water. The product (bromoalkyl acrylate) was extracted with diethyl ether, and washed several times with deionized water. After removal of diethyl ether, the obtained liquid was dried in vacuum at 40 °C. Yield: 70–85%.

In a second step, the bromoalkyl acrylates were mixed with 2.0 equivalents of 1-alkylimidazoles (C_1_Im, C_2_Im, C_4_Im, and C_6_Im) and stirred for 72 h at 50 °C. The received substances were purified by extraction with ethyl acetate and diethyl ether. The products ((acryloyloxy)hexyl alkyl imidazolium bromide, (acryloyloxy)nonyl alkyl imidazolium bromide, and (acryloyloxy)dodecyl alkyl imidazolium bromide) were obtained as yellow, viscous, sticky liquids. Yields: 45–95%. ^1^H NMR, ^13^C NMR: see Supporting Information.

### 2.4. Exchange of Halogen Ions by TFSI

The bromide/iodide anions were exchanged for TFSI anions in the aqueous phase. Initially, the iodide/bromide-ILs were dissolved in deionized water and stirred at 55 °C. After dissolution, the appropriate amount of LiTFSI dissolved in deionized water was slowly added in small molar excess. The obtained solution was stirred at 55 °C for 24 h. During the anion exchange reaction, a phase separation took place since the products with TFSI anions were insoluble in water. They were purified by several washing cycles with deionized water until lithium bromide could no longer be detected with silver nitrate. After drying in vacuum, the monomers were obtained as yellowish to brownish oily room temperature ILs (RTLs). Yields: 80–95%. The anion exchange reaction was confirmed by signal shifts in the ^1^H NMR spectra (e.g., signals of imidazolium group appeared at lower chemical shifts with corresponding TFSI anion instead of bromide anion); see Supporting Information.

### 2.5. Preparation of Polymerized and Crosslinked PIL Films

**Figure 2:**
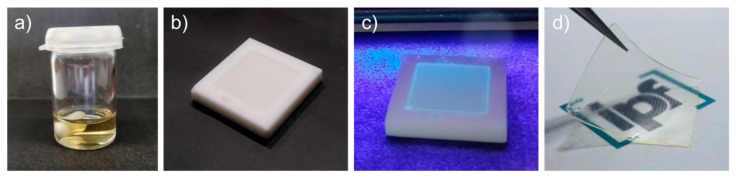
(**a**) Mixture of IL monomer, crosslinker, photoinitiator and LiTFSI; (**b**) liquid mixture in mold; (**c**) UV irradiation and polymerization step; (**d**) polymerized PIL film.

Unless otherwise stated, all monomers were mixed with 5 mol% (with respect to the IL monomer used) crosslinker BAAP, 1 mol% photoinitiator TPO and in selected cases with 10 mol% of lithium conduction salt LiTFSI and stirred for about one hour in the dark before polymerization. The mixtures were then degassed under argon and transferred to a glovebox. There, the mixtures were casted into Teflon forms with specific shape and defined height (2 × 2 cm, 200 µm) and immediately irradiated with UV light (365 nm). After an exposure time of 20 min, the liquid mixtures turned into solid, fairly flexible, and soft, free-standing films, as illustrated in Figure 2, and were carefully removed from the Teflon forms. The thickness of the films was measured on different positions of the sample and averaged values were used. For thickness measurements of the films a Käfer FD50 thickness dial gauge (Käfer Messuhrenfabrik, Villingen-Schwenningen, Germany) with a lightweight lift of contact was used. There was no pressure applied during the thickness measurement to exclude any effect of compression of the samples, resulting in a low measurement failure of about 1 µm due to the lift of contact weight.

### 2.6. Methods

^1^H nuclear magnetic resonance (NMR) spectra were recorded on an Avance III 500 Spectrometer (Bruker Corp. Billerica, MA, USA) at ambient temperature (^1^H NMR: 500 MHz, ^13^C NMR: 126 MHz). For all samples, dimethyl sulfoxide (DMSO-d_6_) was used as solvent. The Raman spectra were recorded on an alpha300R RAMAN Imaging System (WITec GmbH, Ulm, Germany) equipped with a 785 nm laser with a power of 20 mW and a lens with 20× magnification. Polymer samples were investigated after different polymerization times. 200 spectra per sample were recorded and accumulated at an integration time of 0.5 s each. The spectra were normalized to the signal at 747 cm^−1^. The degree of conversion of double bonds was calculated from the decrease in intensity (area integral) of the band at 1660 cm^−1^ relative to the non-polymerized sample:

C_monomer_ = 1 − (I_t_/I_0_)·100% (1)

with C_monomer_ being the monomer conversion, I_0_ being the areal intensity of the band at 1640 cm^−1^ of the non-polymerized sample, and I_t_ the areal intensity of the band at 1640 cm^−1^ after polymerization time t.

Thermogravimetric analyses (TGA) were carried out on a Q5000 (TA Instruments, Newcastle, DE, USA) under nitrogen at heating rates of 10 K·min^−1^ in the temperature range from 30 to 800 °C. Differential scanning calorimetry was performed on a DSC Q2000 (TA Instruments, Newcastle, DE, USA). Samples were subjected to heating-cooling-heating cycles in the temperature range from −80 to 200 °C under nitrogen with heating and cooling rates of 10 K·min^−1^.

The rheological measurements were carried out by means of an ARES G2 rotational rheometer (TA Instruments, Newcastle, DE, USA) using small amplitude oscillatory frequency sweeps and temperature ramps. The selected geometry for frequency sweeps and heating/cooling sweeps was the parallel plate geometry (gap: 1 mm, diameter: 8 mm). All rheological measurements were carried out using nitrogen as the heating gas. Both storage and loss modulus, respectively, were measured as a function of the shear frequency. The complex melt viscosities were calculated. For a number of samples the dependencies of *G′* and *G″* on the temperature were analyzed. The temperature range between −50 and 80 °C was chosen, which covered the temperature range used in electrochemical studies. The samples were first heated to 80 °C and then the cooling run was monitored. Heating and cooling rates were set to 5 K·min^−1^, ω = 5 rad·s^−1^, axial force = 0.5 or 5 N, respectively. Characteristic parameters for the network (effective crosslink density (*ν_eff_*) and average mesh size of the network (L)) were calculated using the plateau modulus obtained in the temperature sweeps according to Equations (2) and (3) according to the relevant literature about network characterization [74,75,76,77].

(2) $$\nu_{eff}=\frac{G^{\prime }}{ART\eta }$$

(3) $$L={\left(\frac{RT}{G^{\prime }N_{A}}\right)}^{\frac{1}{3}}$$

where *G′* is the plateau modulus, *T* the temperature, *R* the gas constant, *N_A_* Avogadro’s number, and *A* the microstructure factor (set to 0.33). *η* is the memory term and indicates the ratio of the end-to-end distance of macromolecules before and after polymerization, and it was assumed that it would not be changed in a photocrosslinking polymerization and therefore set to 1.

### 2.7. Cell Preparation and Electrochemical Characterization

Swagelok cells (Swagelok Co., Solon, OH, USA) were used for ionic conductivity measurements and ECC Standard Cells (El-Cell GmbH, Hamburg, Germany) were used for all other electrochemical measurements. The polymer films prepared as described above were placed between two electrodes. The following setups were utilized for the different electrochemical methods: symmetrical cell setup for complex electrochemical impedance spectroscopy (EIS) and for plating-stripping experiments (Li^0^/PIL/Li^0^); asymmetrical cell setup for linear sweep voltammetry (LSV) or cyclic voltammetry (CV) (Li^0^/PIL/steel). The bottoms and tops of the El-Cell contact stamps were used as steel electrodes. Lithium chips with diameters of 18 mm and 14 mm were supplied by Tob New Energy (Xiamen, China). The thicknesses of the polymer films were determined by a Käfer thickness measurement device. The ionic conductivities were determined by EIS using a VMP3 potentiostat (Biologic, Seyssinet-Pariset, France) within a temperature-controlled climate chamber (CTS, Shanghai Jianheng Instrument Co. Ltd., Shanghai, China). Potentiostatic impedance measurements were carried out with the following parameters: 1 MHz to 100 mHz at open circuit voltage with 25 mV AC current. For the Arrhenius plot, impedance spectra were recorded in the temperature range of 20–80 °C with steps of 10 °C. Before each measurement, the system was equilibrated at the set temperature for 30 min. The ionic conductivity was calculated by Equation (4):

(4) $$\sigma =\frac{d}{RA}$$

with *d* being the sample thickness and *A* being the cross-sectional area of the sample, respectively. The bulk resistance *R* of the polymer materials was determined by fitting model parameters with a suitable equivalent circuit (Figure SI.11) using the Relaxis 3 software (RHD-Instruments, Darmstadt, Germany). Some impedance spectra could not be fitted correctly by Relaxis 3 with the standard model (see Supporting Information). In that case, the bulk resistance was read from the high-frequency intercept of the Nyquist plot with the Z’ real axis. The electrochemical stability was evaluated using cyclic voltammetry measurements (CV). All measurements were carried out by a VMP3 potentiostat in climate chambers (CTS) at a temperature of 30 °C. CV measurements were carried out with 1 mV·s^−1^ scan rate within two different voltage ranges. The first voltage range was from open circuit voltage (OCV) to 5 V vs. Li/Li^+,^ and the second from OCV to 0.05 V vs. Li/Li^+^ to separate anodic from cathodic degradation processes. Lithium plating-stripping experiments were carried out with a VMP3 potentiostat. A constant current was applied to the sample and the voltage change over time was recorded within certain voltage limits. The current was held for one hour each before the polarity was changed. Several current densities were measured to simulate different charge and discharge behaviors for the electrolyte.

(5) $$\sigma =\frac{U}{I_{density}}\cdot d$$

## 3. Results and Discussion

### 3.1. Structural Variations in the PIL Networks

**Figure 3:**
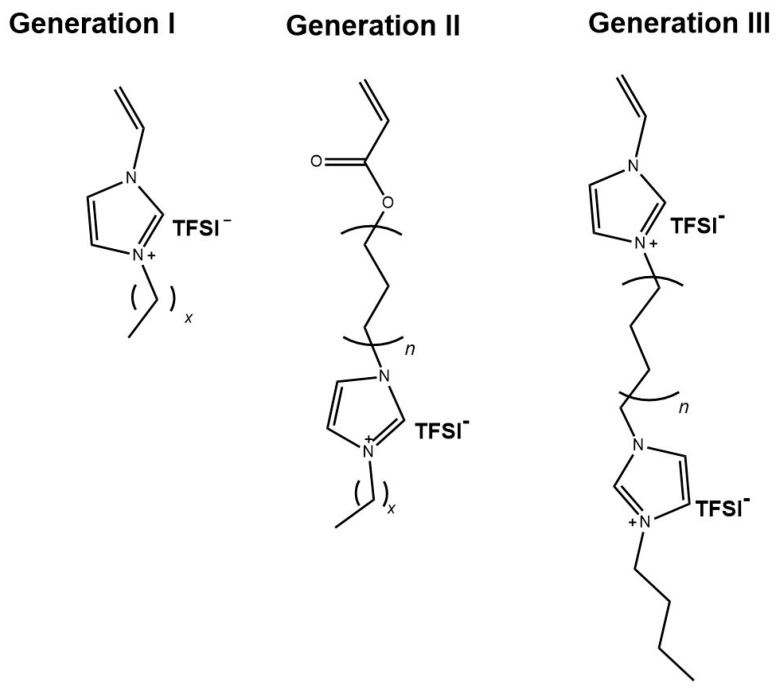
Chemical structures of the IL monomers used for preparation of PIL networks.

The synthetic concept for crosslinked ionic liquid polymers with enhanced ionic conductivity included the preparation of generation II and generation III IL monomers, as illustrated in Figure 3, and the use of different crosslinkers, as shown in Figure 1. All polymer networks were synthesized by UV-initiated free radical polymerization starting from the IL monomers. The chemical structures of the monomers were systematically altered to investigate the influences on the thermal and electrochemical behavior. Initially, 1-alkyl-3-vinylimidazolium TFSI monomers with different alkyl substituents were used (here referred to as generation I, GI). The homopolymers were known from the literature [37]. These polymers (PVImC_x_) were synthesized and polymerized under comparable conditions and served here as the reference. Furthermore, acrylic imidazolium structures were synthesized in which alkyl spacers with different number of methylene groups (C_6_, C_9_, C_12_) were introduced between acrylate group and imidazolium group: This group is referred to as generation II (GII). The monomers of generation III (GIII) were vinylimidazolium compounds with a second imidazolium group which was linked to the first by an alkyl spacer (here referred to as generation III (GIII). The chemical structure of the crosslinker (CL) was varied as well. In the first case with BAAP, a non-ionic structure was chosen, for which additional interactions with the ionic system were not expected. This crosslinker is well-known for fast polymerization under the conditions chosen (room temperature, TPO as initiator, wave length, bulk polymerization) [72,78]. Furthermore, bis(vinylimidazole)s with two ionic units were employed (VIL-C_6_ and VIL-C_12_).

A special feature in this work was the use of in situ polymerization. For this purpose, the method of photopolymerization at room temperature was employed, for which mixtures of monomer, crosslinker, UV initiator, and, in selected cases, conducting salt were irradiated with UV light under inert conditions and polymerized directly into the shape necessary for the measurements. Thus, thin, free-standing polymer films could be produced (see Figure 2 in the experimental section). We note here again that this study focused on the properties of the crosslinked polymer networks containing the IL monomers with different structures and not those of the linear homopolymers (they served only as a comparison). In contrast to the linear homopolymers, crosslinked polymers are not soluble and melt-processable after polymerization, which resulted in restrictions for the chemical characterizations of the materials. Employing monomers and networks prepared under comparable conditions allowed for a reliable comparison between the different structures and direct correlation with network parameters.

### 3.2. Monitoring of the Monomer Conversion in the Crosslinking Free Radical Photopolymerization by Raman Microscopy

**Figure 4:**
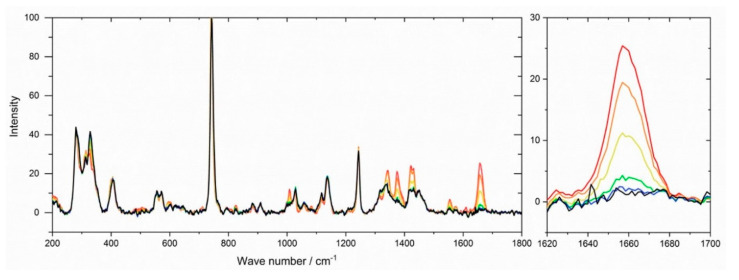
Raman spectra obtained during the UV polymerization of (GI-VC_4_Im TFSI-BAAP)_95:5_ after different reaction times: (red) 0 s; (orange) 30 s; (yellow) 60 s; (green) 120 s; (blue) 600 s; (black) 1800 s. Band of C=C stretching vibration at 1660 cm^−1^ highlighted.

**Figure 5:**
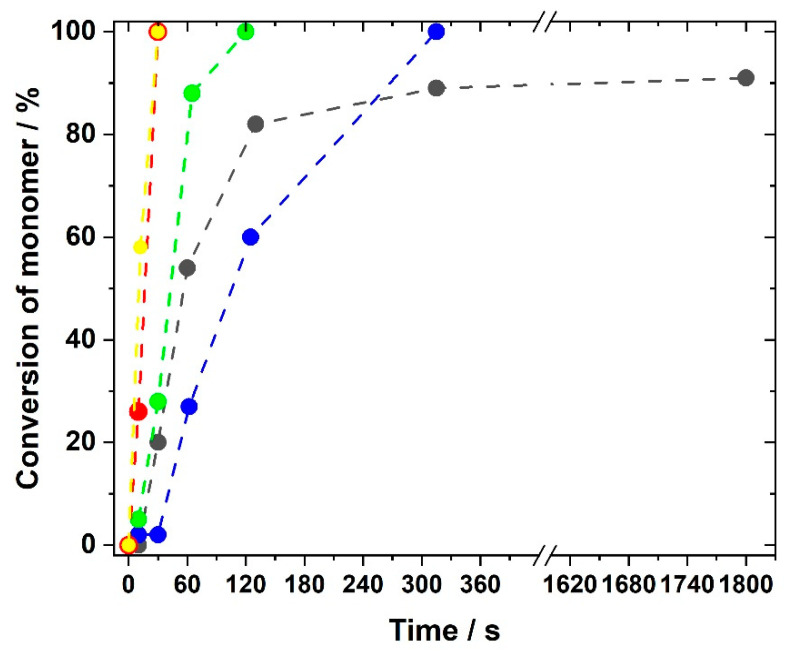
Dependence of the conversion of double bonds on the irradiation time for selected generation I and generation II monomers with 5 mol% crosslinker BAAP and 1 mol% TPO as photoinitiator irradiated with UV light at 365 nm: (black) GI-P(VImC_4_ TFSI-BAAP)_95:5_; (red) GII-P(AAC_6_ImC_4_ TFSI-BAAP)_95:5_; (yellow) GII-P(AAC_6_ImC_4_ TFSI-BAAP)_95:5_ + 10 mol% LiTFSI; (blue) GII-P(AAC_9_ImC_4_ TFSI-BAAP)_95:5_; (green) GII-P(AAC_12_ImC_4_ TFSI-BAAP)_95:5_.

High resolution NMR measurements to confirm the chemical structure were not suitable due to the insolubility of the crosslinked systems. They allowed only determination of whether residual monomers remained in the network without quantification of the conversion. Therefore, the conversion of double bonds during polymerization was monitored by Raman microscopy after different reaction times. Information on the polymerization kinetics to generate IL networks is very seldom in the literature and can only be found for bisacrylate networks with IL plasticizers [70], but not for IL monomers. Figure 4 illustrates Raman spectra obtained during the polymerization of the GI monomer VImC_4_ TFSI with crosslinker BAAP (95:5 mol%:mol%), sample name GI-P(VImC_4_ TFSI-BAAP)_95:5_. The intensity of the band at 1660 cm^−1^ reflecting the stretching vibration of the C=C double bond that is converted into a –C–C– single bond decreased with increasing irradiation time. The conversion-time curves shown in Figure 5 were calculated by the relative intensities of the double bonds.

The influences of both type of generation and spacer length between acrylic group and imidazolium group are clearly visible in Figure 5. The generation I monomer butyl vinylimidazolium TFSI (GI-VImC_4_ TFSI) reacted to a conversion of 80% within two minutes, but the total conversion did not exceed 90%, even after long polymerization times. This value is comparable to data reported by Whitley et al. [79] for the polymerization of mixtures of 1-vinylimidazole with LiTFSI. This is explained by the glass transition temperature T_g_ of the network which was found to be 32 °C for GI-P(VImC_4_ TFSI-BAAP)_95:5_ (the determination of T_g_ is discussed in the next section). With T_g_ above room temperature, the polymerization becomes diffusion-controlled and stops or at least reduces significantly after gelation. For generation II acrylate monomers, the polymerization generally proceeded much faster compared to the vinyl imidazolium compounds of generation I, which was assumed by the comparison of polymerizations of other vinyl and acrylic monomers [65,75]. The fastest monomer conversion was observed for AAC_6_ImC_4_ TFSI, for which the C=C band disappeared after just 30 s and the polymerization could be considered complete. For monomers with the C_9_ and C_12_ spacer respectively, the reaction slowed down, but after five and two minutes, respectively, the polymerization was also complete. The faster polymerization and higher conversion of generation II monomers can again be explained by the T_g_s of the networks: they are well below room temperature, allowing a higher mobility of chains during polymerization. Since use of conducting salts is indispensable for applications in lithium ion batteries, we additionally investigated whether LiTFSI had an influence on the rate of polymerization and the degree of monomer conversion. It was found that the use of conducting salt did not disturb the polymerization rate, as exemplified in Figure 5 for GII-P(AAC_6_ImC_4_ TFSI-BAAP)_95:5_ without (red curve) and with 10 mol% LiTFSI (yellow curve).

For comparison of the polymer samples in the tests, comparable polymerization conditions were chosen for all samples (UV irradiation at 365 nm for 20 min using 1 mol% TPO photoinitiator with respect to the monomer amount).

### 3.3. Glass Transition Temperatures

An important parameter used to describe the thermal behavior of non-structured, amorphous polymer networks is the glass transition temperature (T_g_). The glass transition in the DSC curves (2nd heating curves to eliminate influences of thermal history; see Supporting Information) indicates the temperature range where segmental motions in polymers start [80,81]. In networks, this relates to the behavior of the segments between network knots. Consequently, the T_g_ gives an indirect indication of how flexible a material is at a certain temperature. It is often discussed in the literature that a relationship between T_g_ and ionic conductivity exists [31,32,37]. A lower T_g_ results in higher ionic conductivity because higher flexibility supports ion hopping. The T_g_s of the networks investigated are summarized in Table 1. All DSC curves reflected a well-pronounced glass transition step. The curves can be found in the Supporting Information (Figure SI.1).

A T_g_ of 32 °C was observed for the N-butyl vinylimidazolium-containing polymer network (GI-P(VImC_4_ TFSI-BAAP)_95:5_). Delhorbe et al. [37] found a T_g_ of 3 °C for the linear homopolymer. The difference between a homopolymer and a network is significant even if small variations due to preparation and drying procedures and in the DSC measurement procedures are taken into account. It is reasonable to assume that the networks show higher T_g_s than the corresponding linear polymers due to the reduction of flexibility of the polymer backbone by crosslinking.

For GII polymer networks, T_g_ values of −29 to −42 °C were determined. The T_g_s of networks with IL monomers having comparable alkyl spacer length differed only marginally. The variation of the N-alkyl chain at the imidazolium ring at constant spacer length had a larger effect on T_g_ and reached a T_g_ minimum at −41 °C for GII-P(AAC_6_ImC_4_ TFSI-BAAP)_95:5_. These T_g_ values are significantly higher than values given by Ohno et al. [62] for linear homopolymers using the same heating rate as in this study (10 K·min^−1^). They found, e.g., −69 °C for P(AAC_6_ImC_2_) TFSI.

The addition of conducting salt (LiTFSI) in the in situ preparation of the networks had a weak influence and reduced T_g_ by only a few degrees (in maximum 5 K). The DSC curves are given in the Supporting Information (Figure SI.2).

The introduction of a second imidazolium TFSI group in the structure of the IL monomer in GIII resulted in a significant increase of T_g_ in the GIII networks by 11 to 28 K to higher temperatures than the T_g_s of the corresponding GII networks. The presence of two imidazolium groups per basic unit results in a higher content of ionic clusters, and therefore, a stiffening of the network.

The glass transition temperatures T_g_ were correlated in the following discussion to the rheology results and the ionic conductivities found by EIS.

### 3.4. Thermal Decomposition Behavior

The thermal stability of materials for use in batteries plays a significant role because charge and discharge processes can release large amounts of heat that support the thermal decomposition of the polymers. The thermal decomposition was examined by TGA measurements under nitrogen. The data summarized in Table 1 reveal that all polymer networks were thermally stable in a temperature range up to at least 350 °C when the decomposition started; the maximum decomposition took place between 400 and 426 °C. The detailed TGA curves are given in the Supporting Information in Figure SI.3. The results for T_5%_ and T_max_ were in agreement with literature [82], although here only a one-step decomposition mechanism was observed, whereas the decomposition curves in the literature showed several maxima. We attribute this to the high purity of the monomers used here. The differences in the chemical structures of the monomers (such as spacer length, N-alkyl chain length, and crosslinker) did not influence the thermal decomposition temperatures significantly. A particular trend could not be observed within generations II and III. Addition of LiTFSI enhanced the thermal stability of the materials (TGA curves in the Supporting Information in Figure SI.4). The thermal decomposition maximum of the GIII networks occurred at extremely high temperature (T_max_ at 448–463 °C). An increased number of ionic groups in the polymer network matrix enhances the thermal stability of the materials due to additional ionic binding sites. In summary, it can be noted that the thermal stability of all crosslinked PIL materials was sufficiently high to survive the sometimes harsh conditions in a lithium ion battery.

### 3.5. Determination of the Crosslink Density of the Networks by Rheology

The crosslinked IL networks of generation II in particular were further characterized by their rheological behavior to derive information about the viscosity in the temperature range interesting for ionic conductivities, but also to determine the plateau modulus that can be used to calculate parameters that describe the polymer network (see experimental section). The rubber elasticity theory allows one to determine the network density and the averaged molar mass between crosslinks by using the storage modulus (plateau modulus) [74,75].

**Figure 6:**
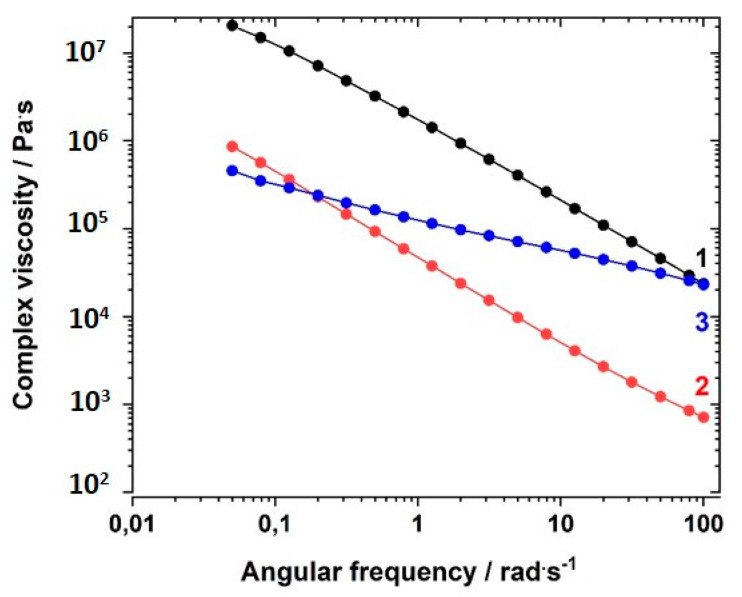
Dependence of the complex viscosity η* on the angular frequency ω at 20 °C for polymers of different generation with comparable crosslinker content at room temperature: (1) GI-P(VImC_4_ TFSI-BAAP)_95:5_; (2) GII-P(AAC_6_ImC_4_ TFSI-BAAP)_95:5_; (3) GIII-P(VImC_6_ImC_4_ TFSI-BAAP)_95:5_.

First, the complex viscosity η* dependence on the angular frequency ω of comparable samples of GI-P(VImC_4_ TFSI-BAAP)_95:5_, GII-P(AAC_6_ImC_4_ TFSI-BAAP)_95:5_, and GIII-P(VImC_6_ImC_4_ TFSI_2_-BAAP)_95:5_ at room temperature (25 °C) was evaluated. Figure 6 shows that the GI network had a higher complex viscosity η* at room temperature than the GII network by at least an order of magnitude. This is caused by the difference in T_g_ (GI: 32 °C vs. GII: −41 °C), and thus, the different states of the samples (glassy vs. viscous). The behavior with increasing shear frequency is comparable. The GIII network depended much less on the shear frequency, which we take as an indication of the formation of an additional ionic network, and of the increased stiffness of the network, as suggested by the enhanced glass transition temperatures. A plateau in the *G′* vs. ω curves was not observed.

**Figure 7:**
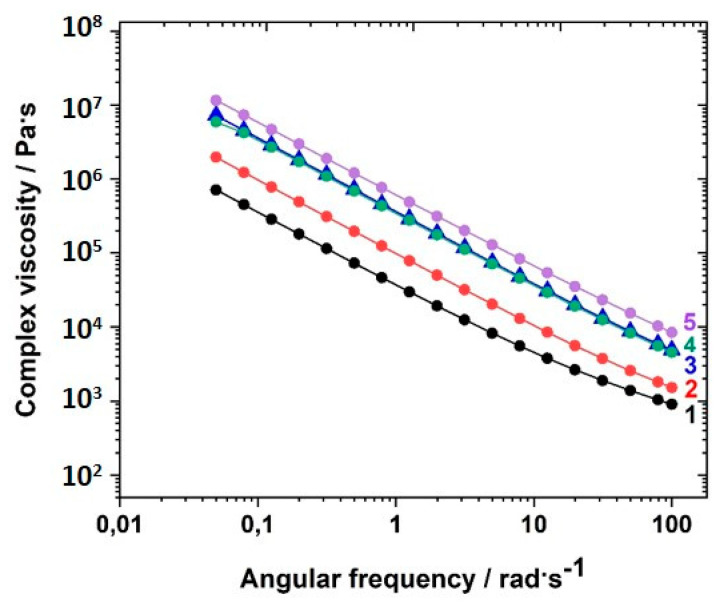
Dependence of the complex viscosity η* on the angular frequency ω at 20 °C for the polymer networks with different amounts of the crosslinker BAAP at room temperature GII-P(AAC_6_ImC_4_ TFSI-BAAP)_x:y_]: (1) 0 mol% BAAP; (2) 2.5 mol% BAAP; (3) 5 mol% BAAP; (4) 7.5 mol% BAAP; (5): 10 mol% BAAP.

The complex viscosities depended on the content of crosslinker used in the network, as illustrated in Figure 7 for the sample series GII-P(AAC_6_ImC_4_ TFSI-BAAP)_x:y_ measured at room temperature. A clear dependence of the complex viscosity on the crosslinker content was observed: the higher the crosslinker content, the higher was the complex viscosity. The difference between the samples was less pronounced at −30 °C (closer to T_g_) (not shown here; see Figure SI.5).

**Figure 8:**
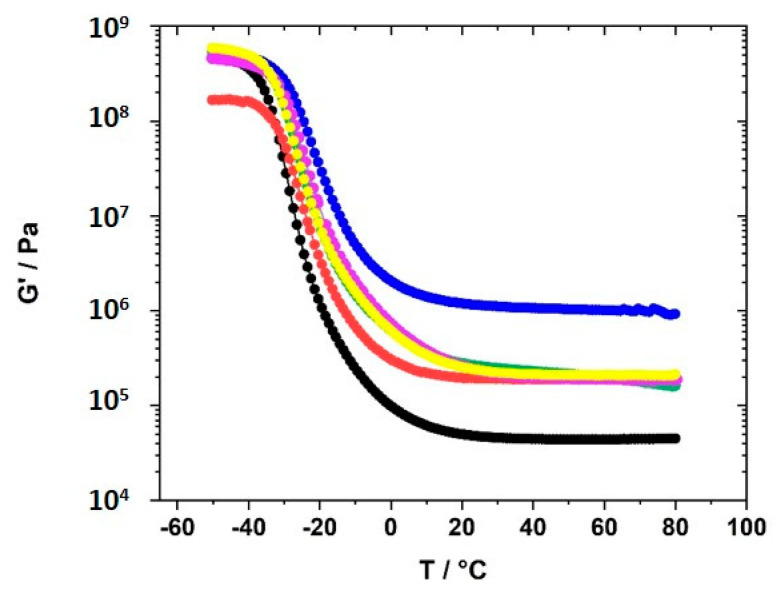
Dependence of the storage modulus *G′* on the temperature for the polymer networks GII-P(AAC_6_ImC_4_ TFSI-BAAP_x:y_ with different amounts of crosslinker BAAP and varying amounts of conducting salt LiTFSI: (black) 0 mol% BAAP; (red) 5 mol% BAAP; (blue) 10 mol% BAAP; (green) 5 mol% BAAP + 10 mol% LiTFSI; (magenta) 5 mol% BAAP + 20 mol% LiTFSI; (yellow) 5 mol% BAAP + 30 mol% LiTFSI.

The curves of *G′* vs. ω did not allow a sound fitting to obtain the plateau modulus (Figure SI.6). Therefore, temperature-dependent measurements were performed to obtain the plateau modulus. The dependence of *G′* on the temperature was measured in the temperature range between 80 °C and −50 °C after heating the samples to 80 °C. The cooling runs after heating the samples to 80 °C were monitored. This measuring range covers the range in which electrochemical measurements were taken. Figure 8 displays the *G′* vs. temperature curves of GII-P(AAC_6_ImC_4_ TFSI-BAAP)_x:y_ samples with varying content of crosslinker BAAP and varying content of LiTFSI conducting salt.

The plateau modulus was determined as *G′* value at 60 °C, because for all samples *G′* at that temperature reached the plateau and no strong deviations were observed. The values were taken directly from the graphs. The effective crosslink density *ν_eff_* and the average mesh size L between network nodes was calculated using *G′* (60 °C). The values are summarized in Table 2.

**Figure 9:**
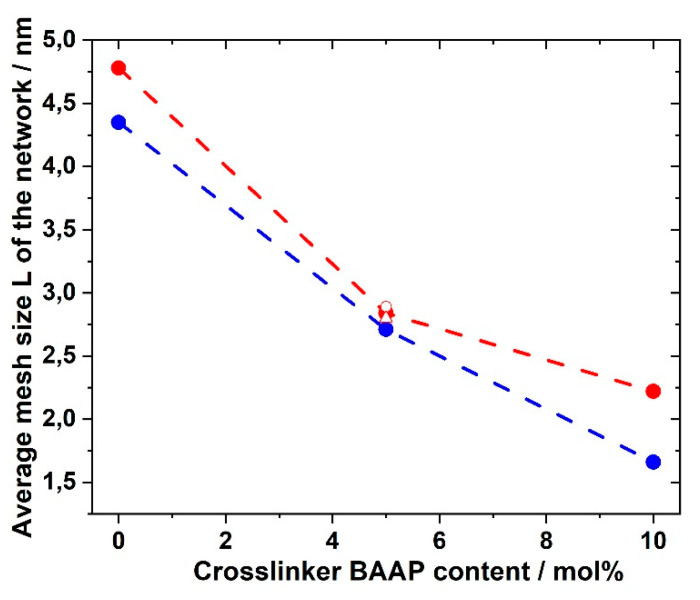
Average mesh size L of GII-P(AAC_6_ImC_4_ TFSI-BAAP)_x:y_ networks depending on the crosslinker BAAP content: (blue) networks without LiTFSI; red: networks with 10 mol% LiTFSI; (red open circle) 20 mol% LiTFSI; (red open triangle) 30 mol% LiTFSI.

The plateau modulus *G′* increased with increasing amount of crosslinker BAAP, as illustrated in Figure 8, which directly influenced the network parameters. Consequently, the average mesh size L of the networks without LiTFSI decreased from 4.35 nm without crosslinker (which is only a formal value because a covalent network did not exist) to 1.66 nm in the network with 10 mol% crosslinker (Table 2). Addition of 10 mol% LiTFSI enhanced L in the networks slightly, which can be attributed to the incorporation of TFSI ions with an average diameter of about 0.724 nm, as calculated by Nilsson-Hallén et al. [66] (own calculations by molecular dynamics with geometry optimization yielded 0.792 nm). A further increase in the LiTFSI content in the samples with 5 mol% crosslinker did not have further effects on L, as illustrated in Figure 9. The trends for the effective crosslink density *ν_eff_* followed the inverse tendency, as could be expected (compare Table 2).

The glass transition temperatures T_g,tan__δ_ obtained from the maximum in the tanδ curves (Figure SI.7) were about 20 K higher than the values found by DSC, which was mainly due to the different heating rate (5 vs. 10 K·min^−1^ in DSC, respectively), but also due to the experimental conditions. However, these values (see last column in Table 2) revealed that the difference between the linear homopolymer sample (entry 1) and the crosslinked polymers (entries 3 and 4) is not significantly high (only 7 K), and lower than assumed from the comparison with DSC T_g_ results, and from the values given by Ohno [62] for the homopolymer, for which the difference was more than 40 K. Increasing the amount of crosslinker altered T_g,tan__δ_ very weakly, similarly to what was shown by the values obtained by DSC. The same was noted after addition of LiTFSI. Even significant amounts of LiTFSI (30 mol%, entry 8) did not change the T_g_, and thus the dynamics of the backbone. Comparable trends are reflected by the *G′* vs. T curves in Figure 8. Increasing amounts of crosslinker enhanced the storage modulus *G′* and restricted the mobility.

### 3.6. Ionic Conductivity of the PIL Networks

The main focus of this work was to investigate the influence of chemical structure of the monomer used in the PIL network on the ionic conductivity of the resulting materials. The goal was to introduce higher chain flexibility into monomers of generation I in order to achieve higher ionic conductivities σ (we assumed that a correlation between T_g_ and σ exists, as stated in the literature [37,63]). Spacer length, N-alkyl chain length, number of ionic groups, crosslinker type and content, and the amount of conducting salt were varied. The ionic conductivity was determined in the temperature range between 20 and 80 °C by means of EIS and calculated from the bulk resistance R_b_ measured (see experimental section). Illustrating examples for Nyquist plots are given in the Supporting Information (Figure SI.9, Figure SI.10). Figure SI.11 depicts the equivalent circuit used for the calculations of σ. The equivalent circuit contained more elements than in examples of gel electrolytes with liquid electrolytes [83,84,85] due to the higher number of interfaces in the system and the fact that the ion conduction mechanism in liquids is different to that in solid state electrolytes in the present system.

#### 3.6.1. GI Networks

The properties of GI homopolymers have been reported in detail in the literature [37]. Polymers were synthesized under conditions used in this study and the generation I butyl vinylimidazolium network GI-P(VImC_4_ TFSI-BAAP)_95:5_ served as the reference. The ionic conductivity of this material was found to be 5.09 × 10^−9^ S·cm^−1^ at 30 °C and reached 5.93 × 10^−6^ S·cm^−1^ at 80 °C. The values for the network GI-P(VImC_4_ TFSI-BAAP)_95:5_ were 0.5 to 1 orders of magnitude lower than the results reported before [37]. We attribute this to the existence of the PIL network in contrast to the linear homopolymers and the restriction of mobility by network formation.

#### 3.6.2. Influence of Spacer Length in GII and GIII Networks

**Figure 10:**
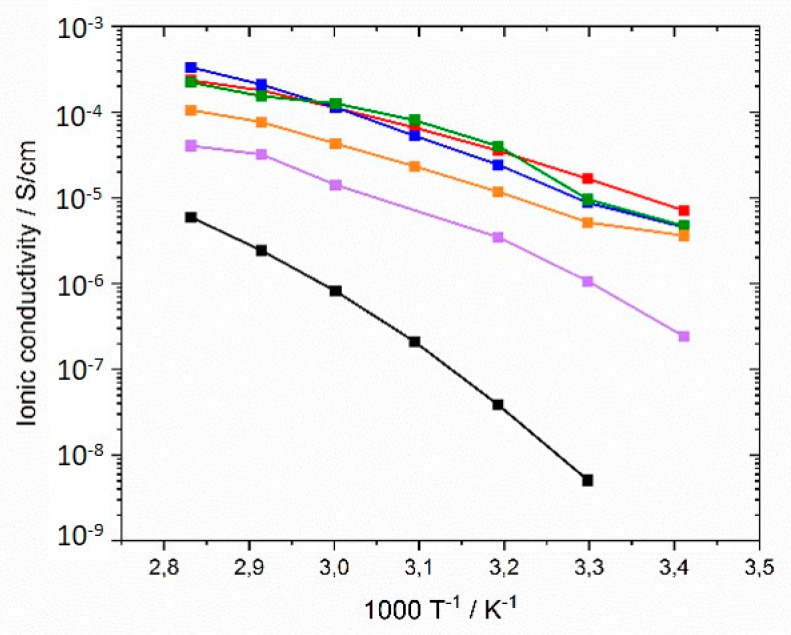
Ionic conductivities of PIL networks from monomers with different alkyl spacer lengths in the temperature range between 20 and 80 °C: (black) GI-P(VImC_4_ TFSI-BAAP)_95:5_; (red) GII-P(AAC_6_ImC_4_ TFSI-BAAP)_95:5_; (blue) GII-P(AAC_9_ImC_4_ TFSI-BAAP)_95:5_; (green) GII-P(AAC_12_ImC_4_ TFSI-BAAP)_95:5_; (violet) GIII-P(VImC_6_ImC_4_ TFSI-BAAP)_95:5_; (orange) GIII-P(VImC_12_ImC_4_ TFSI-BAAP)_95:5_.

In the next step, an alkyl spacer (C_6_, C_9_ and C12) was inserted between polymer backbone and imidazolium group according to the concept described by Ohno et al. [62,63,64]. Figure 10 shows the comparison of ionic conductivities of generation I, generation II, and generation III networks with comparable crosslinker type and concentration. It was noted that higher ionic conductivities than for generation I networks were achieved over the entire temperature range. The GII-PIL networks reached values between 1.03 × 10^−5^ S·cm^−1^ at 20 °C and 3.5 × 10^−4^ S·cm^−1^ at 80 °C. The transition from generation I to generation II achieved two to three orders of magnitude higher ionic conductivity.

No influence of spacer length between the main chain and imidazolium group was not found in the networks consisting of comparable monomers, as illustrated in Figure 10, in contrast to that described by by Yoshizawa et al. [63] for linear homopolymers. While the authors reported increasing ionic conductivity with increasing spacer length, we observed conductivity values in the networks with C_9_ (sample GII-P(AAC_9_ImC_4_ TFSI-BAAP)_95:5_) and C_12_ (sample GII-P(AAC_12_ImC_4_ TFSI-BAAP)_95:5_) spacers similar to those with the C_6_ spacer (sample GII-P(AAC_6_ImC_4_ TFSI-BAAP)_95:5_). Again, the crosslinking appeared to be the limiting factor for the ion mobility. This is a strong indication that the ion mobility in the networks is not decoupled from the segmental dynamics of the polymer matrix, as has been discussed recently for linear PILs with long alkyl spacers in the side chain.

**Figure 11:**
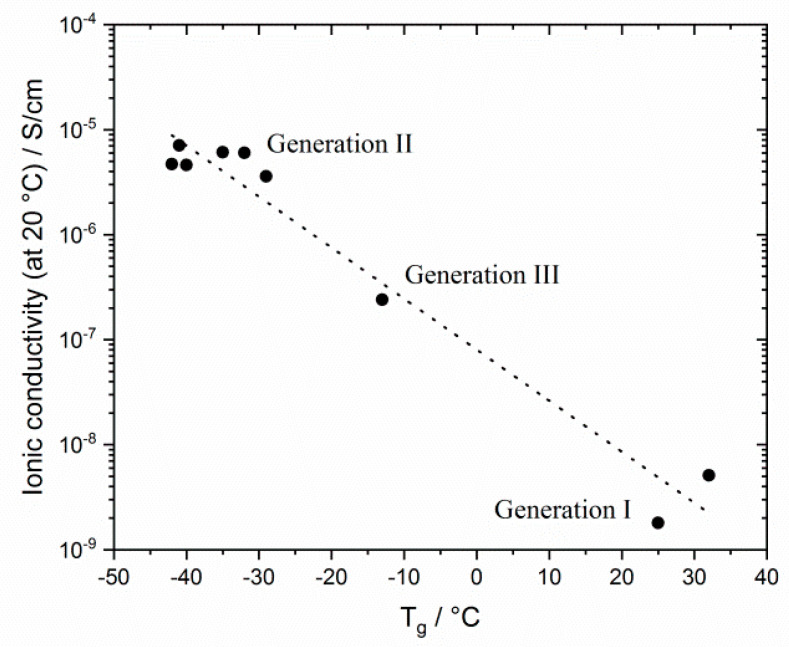
Correlation between glass transition temperature T_g_ and ionic conductivities σ at room temperature for selected PIL networks studied (with 5 mol% BAAP as crosslinker).

The results reflect that T_g_ is a determining factor for ionic conductivity: The T_g_s of generation II networks are significantly lower than those of generation I. Generation III networks had higher ionic conductivities than for generation I, but lower ionic conductivities than for generation II networks were obtained over the whole temperature range. This finding correlates to the behavior of the T_g_s. Linear homopolymers had a slightly higher ionic conductivity than networks (compare also Figure 13). In contrast to generation II polymer networks, the alkyl spacer length played a significant role in the ionic conductivity of GIII networks. The GIII-PIL network with C_6_-spacer (GIII-P(VImC_6_ImC_4_ TFSI-BAAP)_95:5_) reached values of 2.4 × 10^−7^ S cm^−1^ at 20 °C and 4.0 × 10^−5^ S cm^−1^ at 80 °C. The GIII-PIL network with C_12_-spacer (GIII-P(VImC_12_ImC_4_ TFSI-BAAP)_95:5_), on the other hand, achieved noticeably higher values of 3.6 × 10^−6^ S·cm^−1^ at 20 °C and 1.0 × 10^−4^ S·cm^−1^ at 80 °C than the GIII network with the shorter spacer unit. Figure 11 visualizes the dependence of the ionic conductivity of the PIL networks on the T_g_ of networks from different generation.

Since the GII network with the C_6_ spacer GII-P(AAC_6_ImC_4_ TFSI-BAAP)_95:5_ showed suitable ionic conductivity and the preparation procedure was quite convenient, all further investigations were performed with that monomer.

#### 3.6.3. Influence of N-alkyl Length in Generation II Monomers

In addition to the alkyl spacer length positioned in the center of the monomer, the length of the N-alkyl side groups on the imidazolium ring was varied in generation II monomers among C1, C2, C4, and C6. The influence of these N-alkyl groups was not significant, although the T_g_ values given in Table 1 suggested a different behavior. The curves σ vs. 1/T are given in the Supporting Information (Figure SI.12). The main difference was found in the room temperature range, where the network GII-P(AAC_6_ImC_1_ TFSI-BAAP_95:5_) with the monomer with the N-methyl chain showed the lowest ionic conductivity with 3.6 × 10^−6^ S cm^−1^ at 20 °C. A slight increase with ethyl in GII-P(AAC_6_ImC_2_ TFSI-BAAP_95:5_) and hexyl group in GII-P(AAC_6_ImC_6_ TFSI-BAAP)_95:5_ and a maximum with 7.1 × 10^−6^ S cm^−1^ at 20 °C in GII-P(AAC_6_ImC_4_ TFSI-BAAP)_95:5_ were found. In contrast to these results, Delhorbe et al. [37] found that the ionic conductivity of linear generation I polymers raised with increasing N-alkyl chain length up to a chain length of C_6_ and then decreased again. A plasticizing effect of the alkyl side chain was discussed.

#### 3.6.4. Influences of Crosslinker Type and Content Compared to Linear Homopolymers

**Figure 12:**
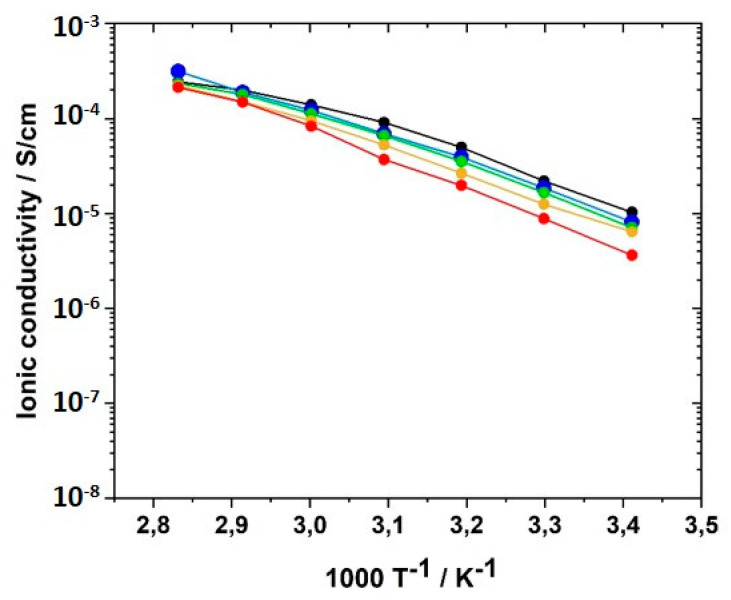
Influence of crosslinker (BAAP) concentration on the ionic conductivity of GII PIL networks with the monomer GII-AAC_6_ImC_4_ TFSI in the temperature range from 20 to 80 °C: (black) 0.0 mol%; (blue) 2.5 mol%; (green) 5.0 mol%; (yellow) 7.5 mol%; (red) 10.0 mol%.

To further elucidate the role of the crosslinker BAAP with respect to the ionic conductivity, the crosslinker content was varied from 0 to 10 mol% with respect to the IL monomer in 2.5 mol% increments and the ionic conductivity of the networks was determined. σ of the linear homopolymer GII-P(AAC_6_ImC_4_ TFSI) was found to be 1.03 × 10^−5^ S·cm^−1^ at 20 °C, and 2.43 × 10^−4^ S cm^−1^ at 80 °C, values that were in good agreement with those published by Ohno et al. [62]. In Figure 12, the ionic conductivity is shown as a function of temperature for different BAAP crosslinker contents. The differences between the samples were more pronounced at room temperature (closer to T_g_ than the other measuring temperatures).

**Figure 13:**
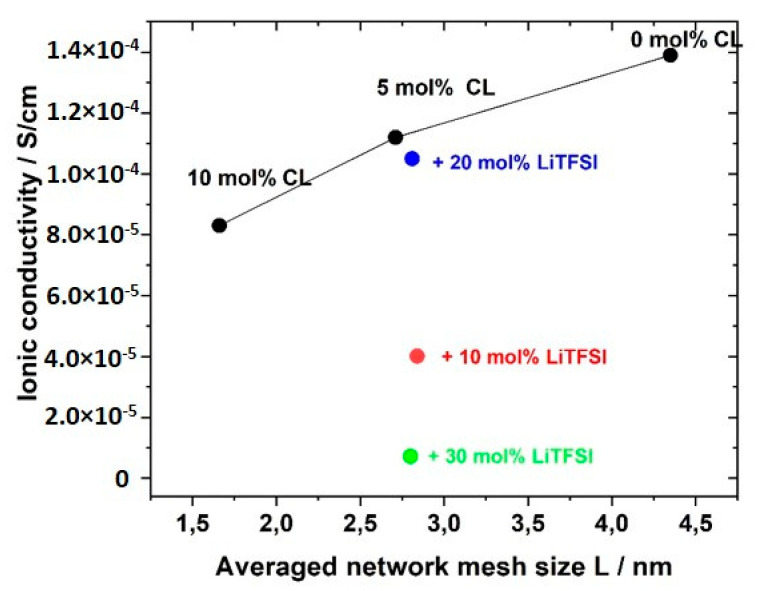
Influence of average mesh size L of the networks on the ionic conductivity of GII PIL networks with the monomer GII-AAC_6_ImC_4_ TFSI without (black) and with LiTFSI conducting salt (blue: 20 mol% LiTFSI; red: 10 mol% LiTFSI; green: 30 mol% LiTFSI).

The ionic conductivity dropped slightly with increasing crosslinker content; that meant, reduced averaged mesh size L between the network nodes, as visualized in Figure 13. The stiffness of the network increased with crosslinking content and thus reduced ion transport and ionic conductivity. The network nodes may also act as a mechanical barrier for the ion movements. The addition of the conducting salt also reduced the ion conductivity.

**Figure 14:**
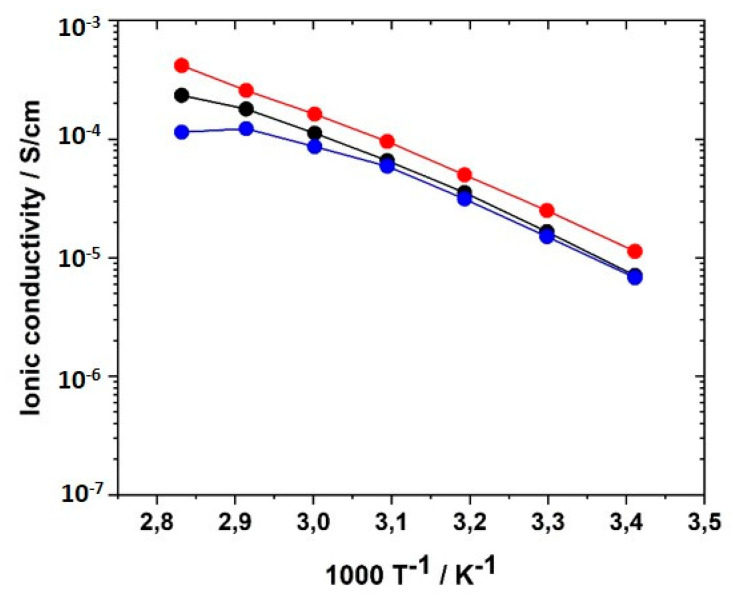
Influence of crosslinker type on the ionic conductivity of networks with the monomer GII-AAC_6_ImC_4_ TFSI: (black) 5.0 mol% BAAP; (red) 5.0 mol% VILC_6_; (blue) 5.0 mol% VILC_12_.

Another reason for the reduced ion conductivity compared to linear homopolymers could be the decreased concentration of ionic sites due to the use of the non-ionic crosslinker BAAP. To investigate the influence of the crosslinker type, the ionic crosslinkers VIL-C_6_ and VIL-C_12_ were synthesized and employed with the generation II monomer GII-(AAC_6_ImC_4_ TFSI). Figure 14 illustrates that VIL-C_6_ enhanced σ slightly over the whole temperature range examined, but this weak advantage may not be worth it looking into, considering the synthetic efforts for the synthesis. VIL-C_12_ was expected to provide an even larger ion permeability, but no relationship was observed.

It is clearly visible that crosslinking resulted in a reduction of ion conductivity compared to the linear polymer analogues. However, it has to be pointed out that the PILs, besides ion conduction, also have to perform as separators between the electrodes in solid-state batteries. This function requires a certain mechanical stability of the polymer electrolyte (PEL) and can be provided best in acrylic low T_g_ polymers by crosslinking. Consequently, a compromise between ionic conductivity, mechanical stability, and handling (in terms of stickiness and brittleness) has to be found. For this reason, a crosslinker content of 5 mol% was chosen for all further investigations in this work.

#### 3.6.5. Influence of Conducting Salt

**Figure 15:**
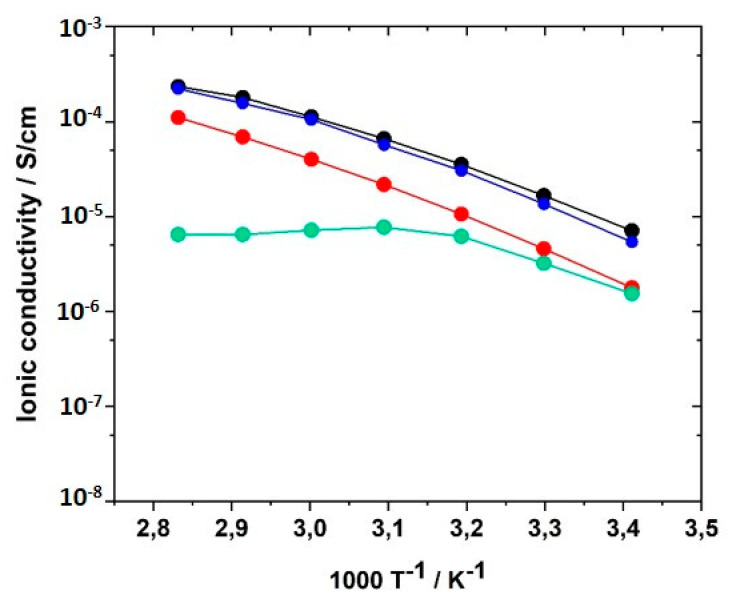
Influence of the amount of conducting salt LiTFSI on the ionic conductivity of GII-P(AAC_6_ImC_4_ TFSI-BAAP_95:5_) + x mol% LiTFSI networks in the temperature range between 20 and 80 °C: (black) x = 0 mol%; (red) x = 10 mol%; (blue) x = 20 mol%; (green) x = 30 mol%.

For potential application of the PILs in lithium ion batteries, the addition of conducting salt is indispensable to providing mobile lithium ions to the system because the lithium ion concentration plays a significant role for σ. Therefore, mixtures of the monomers with concentrations of conducting salt (LiTFSI) varying between 0 to 30 mol% were prepared and UV-polymerized. LiTFSI contents of more than 30 mol% could not be realized due to limited solubility in the monomer formulation. Figure 15 illustrates the influence of the LiTFSI concentration on the ionic conductivity of PIL networks GII-P(AAC_6_ImC_4_ TFSI-BAAP)_95:5_. LiTFSI concentrations of 10 and 30 mol%, respectively, led to the reduction of ionic conductivity by half an order of magnitude at 20 °C. The drop was even more pronounced for 30 mol% LiTFSI in the temperature range between 40 and 80 °C. A possible explanation for the reduction is the formation of stable complexes of TFSI and lithium ions triggered by coulombic interactions [86,87,88,89]. Such complexes would reduce the mobility of the ions. With 20 mol% LiTFSI added, the ionic conductivities reached the values obtained without conducting salt; i.e., an additional enhancement of the conducting salt in terms of σ could not be observed. Change of the anion used in the monomers by substitution of TFSI^−^ by PFSI^−^ ([N(C_2_F_5_SO_2_)_2_]^−^, (bis(pentafluoroethanesulfonyl)imide), or perchlorate ClO_4_^−^ yielded a reduction of ion conductivities.

### 3.7. Lithium Plating-Stripping Experiments

Before using the polymer electrolyte in a cell with electrodes, the ion transport ability had to be further evaluated. Impedance spectroscopy provides information about the bulk lithium ion conductivity, but gives only limited information about the ion transport through the polymer material and through interfacial layers between electrode and electrolyte/separator. In lithium plating-stripping experiments, the ions move from the metallic lithium electrode through the polymer and are plated on the other metallic lithium electrode. On their path, the ions have to cross the material interfaces between polymer electrolyte and metallic lithium electrodes. With the plating-stripping experiment, the total resistance of the symmetrical setup can be studied. A constant current was applied and the voltage was measured as function of time. The current polarized the sample, and anions and cations were separated and moved to the electrodes.

**Figure 16:**
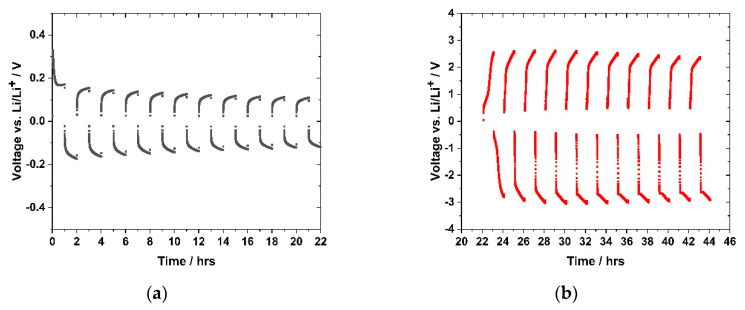
Lithium plating-stripping experiment with sample GII-P(AAC_6_ImC_4_ TFSI-BAAP_95:5_) + 20 mol% LiTFSI) with two different current densities (1.27 and 12.7 μA cm^−2^) at room temperature in the voltage range between −5 amd 5 V, for one hour before current direction change: (**a**) cycling experiment with a current of 1.27 μA cm^−2^; (**b**) cell measured in (**a**), subsequently cycled with a current of 12.7 μA cm^−2^.

For those experiments, the sample (GII-P(AAC_6_ImC_4_ TFSI-BAAP_95:5_) + 20 mol% LiTFSI) was selected. Constant currents of 1.27 μA cm^−2^ and 12.7 μA cm^−2^, respectively, were applied. The current direction was changed ten times (referred to as cycling). A steady current was measured after the initial polarization phase, which is attributed to the flow of lithium ions through the polymer electrolyte in the symmetric cell (lithium/PIL network/lithium). Figure 16a shows the results obtained in the cycling with 1.27 µA cm^−2^, and Figure 16b shows the cycling with 12.7 µA/cm^2^, respectively. A significant difference in the sample behavior with the different current densities was observed. The potential for 12.7 μA cm^−2^ was in the range of (+/−) 2.5–3.0 V vs. Li/Li^+^, whereas the voltage observed at the lower current density of 1.27 µA cm^−2^ ranged between (+/−) 0.2 V vs. Li/Li^+^. In the early stage of the measurement (Figure 16a), a fast increase of the voltage to 0.3 V followed by a rapid decrease to 0.17 V was detected. It attributed to an initial polarization effect before the lithium ion current started. In the second cycle, the voltage initially rose and approached the value of 0.19 V just after applying the current of 1.27 μA cm^−2^. After changing the current direction, a voltage of −0.17 V was reached. After ten cycles, the positive and negative voltages reached 0.10 V and −0.12 V, respectively. This indicated a slightly higher resistance on the negative side, but no voltage, and thus, resistance increase over the cycles.

Figure 16b demonstrates the Li plating-stripping behavior of the sample after raising the current density to 12.7 μA cm^−2^. The potential immediately reached 2.5 V, but a plateau comparable to the one in Figure 16a was not observed. In the first cycle a positive voltage of 2.5 V and a negative voltage of −2.8 V was attained. The values increased until the fourth cycle to 2.6 V and −3.0 V, respectively, and afterwards reduced values were noted. Within the measuring time, all cycles did not show a steady state voltage profile, as found at 1.27 μA·cm^−2^. Altering the current density to 12.7 μA cm^−2^ affected this phenomenon even more. The strong voltage increase indicated that a voltage plateau was far away from being reached. The ionic conductivities calculated from the resistance values obtained in the lithium plating-stripping experiments are summarized in Table 3.

By comparing the specific ion conductivity values with the bulk ion conductivities calculated from EIS measurements (compare Figure 10), it was noted that the values obtained by EIS were two orders of magnitude higher than the values from lithium plating-stripping experiments. Sample (GII-P(AAC_6_ImC_4_ TFSI-BAAP)_95:5_ + 20 mol% LiTFSI showed a bulk conductivity of 5 × 10^−6^ S·cm^−1^, whereas the specific conductivity from plating−stripping experiments was 8 × 10^−8^ S·cm^−1^. As explained above, the resistance of the complete setup was determined in these measurements, which was higher than the bulk resistance of the PIL in EIS measurements. Thus, the specific conductivity calculated from the 2nd semicircle of sample GII-P(AAC_6_ImC_4_ TFSI-BAAP)_95:5_ + 20 mol% LiTFSI with a resistance of 9878 Ω resulted in a value of 4.8 × 10^−7^ S/cm (Figure SI–10), which matches the specific conductivity of 1.54 × 10^−7^ S/cm at 1.27 µA/cm^2^.

The results demonstrated that reversible lithium plating-stripping processes occurred at low current density (1.27 μA·cm^−2^) at 30 °C. During cycling, the voltage remained constant; i.e., blocking interface layers or degradation products were not formed. After increasing the current density by a factor of 10, these processes resulted in a higher voltage by a factor of approximately 10, which means the ion conductivity remained in the same order of magnitude. As the ionic bulk conductivity differed by two orders of magnitude from the value calculated from the plating-stripping experiments it was concluded that the interface between PIL and electrodes plays a crucial role. The specific conductivity values obtained from the 2nd semicircle matches the specific conductivity values from the plating stripping experiment. Future experiments will cover this question and aim to improve interface boundaries between electrodes and the polymer electrolyte to ensure cyclability in a lithium battery cell design with a cathode.

### 3.8. Voltage Stability

**Figure 17:**
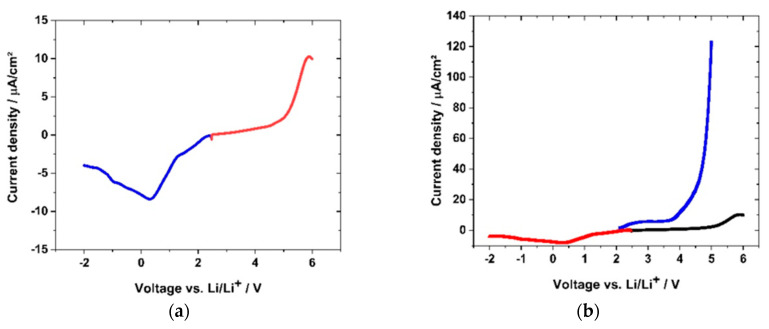
LSV stability test (with 1 mV·s^−1^) vs. lithium of two separate samples of GII-P(AAC_6_ImC_4_ TFSI-BAAP_95:5_ + 20 %mol LiTFSI and PEO-LiTFSI polymer electrolyte: (**a**) (red) positive voltage region; (blue) negative voltage region of GII-network; (**b**) LSV curve scaled in comparison to (black) (PEO + 25 %mol LiTFSI) polymer electrolyte.

For the function of the polymer electrolyte in a cell setup with an anode and cathode, it is crucial to evaluate the oxidation and reduction stability at the different electrode potentials. If an electrochemical induced oxidation or reduction is detected, the prerequisite of the function of the polymer electrolyte might be endangered. The electrode potential will be reached hundreds of times within battery charge and discharge cycles, which can finally degrade the electrolyte and lead to cell failure. Therefore, the electrochemical stability towards oxidation and reduction was tested by means of linear sweep voltammetry (LSV). Two different voltage ranges were examined: first, the range of 2.0 V and 5.0 V, and second, the range of −2.0 V to 2.0 V vs. Li/Li^+^. The red curve in Figure 17a shows the stability of sample GII-(P(AAC_6_ImC_4_ TFSI-BAAP)_95:5_ + 20 %mol LiTFSI) towards the high voltage region forcing an anodic oxidation reaction. The current increase in Figure 17a followed an approximately exponential trend, approaching the maximum oxidation current at 5.9 V vs. Li.

The current density reached a value of 0.01 mA·cm^−2^ at 5.0 V and a value of 8.6 × 10^−4^ mA·cm^−2^ at 4.3 V. In the anodic region, a strong decrease of the current density was measured, reaching the minimum (8.3 × 10^−3^ mA·cm^−2^) at 0.35 V. At −1.0 V vs. Li/Li^+^, the current density reached −6.0 × 10^−3^ mA·cm^−2^. Although the curve in the negative region showed a steep increase and reaction peak, the values obtained were far below these of the polymer electrolyte (PEO + 25 %mol LiTFSI) prepared according to the literature [24]. PEO electrolytes are described as unstable in voltage ranges above 4 V, except special crosslinked samples with short PEO segments [90]. Literature LSV measurements of room temperature ILs reached a current density below 0.1 mA·cm^−2^ in the stable state [91]. Figure 17b displays the LSV measurement of the GII-(P(AAC_6_ImC_4_ TFSI-BAAP)_95:5_ + 20 %mol LiTFSI network normalized to the PEO-LiTFSI PIL degradation. Compared to the degradation reaction observed there, the degradation of the GII-network above 4 V was two orders of magnitude lower in terms of current density.

**Figure 18:**
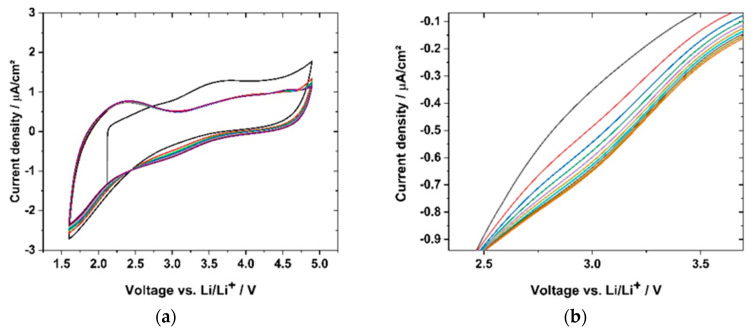
(**a**) CV scan (1 mV·s^−1^) of sample GII-P(AAC_6_ImC_4_ TFSI-BAAP_95:5_) + 20 mol% LiTFSI) within battery electrode-relevant voltage ranges; (**a**) from cycle 1 (black) to cycle 10 (red); (**b**) zoom-in of the anodic peak at 3.0 V, showing the reduction of current density with increasing cycle number.

Cyclic voltammetry (CV) was used to evaluate the cycling stability. The samples were cycled within the relevant voltage region of a full cell with LiNi_0.5_Mn_1.5_O_4_ (LNMO) and Li_4_Ti_5_O_12_ (LTO) electrodes. Figure 18a shows the CV cycling of sample GII-P(AAC_6_ImC_4_ TFSI-BAAP)_95:5_ + 20 mol% LiTFSI within the potential window of 4.9 V and 1.6 V vs. Li/Li^+^. Reversible cycling behavior within 10 cycles with 1 mV·s^−1^ was observed. The flat parts of the CV plot reflect capacitance currents (e.g., 3.5–4.5 V for negative current). In the cathodic region one broad major peak at 2.4 V and two small, broad peaks (at 3.9 and 4.6 V) were detected. We ascribe these peaks to degradation reactions. As all the peaks mentioned decreased to below 1 μA·cm^−2^, and subsequently, the reactions were not considered critical for the function of the polymer electrolyte. In the anodic scan one broad peak with a maximum at 3.0 V appeared. It should be noted that the current densities of the cathodic peaks in all cycles were lower than in the first cycle. The origin of the peaks might be traces of halogen ions which might have been present due to the synthesis route. Figure 18b shows the magnification of the broad peak at 3.0 V, where a slight increase in current density was observed and thus an increase of the peak height was detected. With regard to the peak increase over 10 cycles, the difference of the current densities with regard to the previous cycle was decreasing, meaning the peak was increasing with less intensity. Thus, further cycling may not lead to a significant peak increase.

In conclusion, the CV diagrams showed a reversible reaction of the sample (P(AAC_6_ImC_4_ TFSI-BAAP)_95:5_ + 20 mol% LiTFSI), revealing more capacitance effects than significant degradation reactions of the polymer. This supports the findings from the lithium plating-stripping experiment, wherein no degradative layers were formed during cycling. Furthermore, the CV cycles support the LSV curve, indicating a negligible current increase up to 5.0 V vs. Li/Li^+^, as illustrated in Figure 18.

## 4. Conclusions

In this study, we presented the synthesis, photopolymerization, and detailed characterization of crosslinked imidazolium-based poly(ionic liquids) with monomer chemical structures altered stepwise (generation I-III), among them a number of new IL monomers. The GIII monomers, being bis(vinylimidazolium) TFSI salts with two ionic sites per repeating unit, have not been previouyly reported. The procedure using UV-initiated crosslinking polymerization of mixtures of monomer, crosslinker, and conducting salt offers a facile and fast method with high degrees of conversion. Crosslinking provided enhanced mechanical stability compared to linear homopolymers, resulting in free-standing films that can also be used as separators. The averaged mesh size of the PIL networks can be controlled by the amount of crosslinker used and relates directly to the ionic conductivity.

The series of samples prepared under exactly controlled conditions with carefully purified monomers allowed for the first time a direct comparison of PILs based on different monomer systems polymerized as linear homopolymers and networks. Thus, more general statements could be exemplified by samples analyzed also under comparable conditions. The influence of the monomer structure (GI vs. GII vs. GIII) on the ionic conductivity was demonstrated. In comparison to well-known poly(vinylimidazolium TFSI) materials (generation I), GII and GIII-PIL reached values of ionic conductivity which were improved by several orders of magnitude. The results revealed that parameters such as additional spacers, the number of imidazolium groups, the conducting salt, and type and content of crosslinking have an enormous effect on the ionic conductivity. The highest values of ionic conductivity that could be reached with these materials were in the range of 7.1 × 10^−6^ S·cm^−1^ at 20 °C and 2.3 × 10^−4^ S·cm^−1^ at 80 °C, respectively. The transition from linear homopolymers to networks reduced the ionic conductivity by about one order of magnitude, but allowed free-standing films instead if sticky materials. Thus, a careful tradeoff of desired properties must be considered.

We showed that the PIL networks have a much higher voltage stability than PEO electrolytes with the same amount and type of conducting salt (LiTFSI). GII-PIL networks are electrochemically stable up to a potential of 4.7 V vs. Li/Li^+^, which is crucial for a potential application as a solid electrolyte. Cycling (CV and lithium plating-stripping) experiments revealed that it is possible to conduct lithium ions through the GII-PIL networks at low currents. These experiments in particular showed the importance of the interface between electrode and polymer electrolyte for the ion transition between the materials. Further investigation of interface processes would allow improvements of the system. They also showed high thermal stability and did not decompose below 300 °C, a temperature range sufficiently high for applications in electrochemical cells. In summary, the obtained results revealed that the GII-PIL materials presented could be potential candidates for an application as a solid-state polymer electrolyte in lithium ion batteries.

## Figures and Tables

**Table 1:** Thermal characteristics of synthesized PIL networks with comparable crosslinker and crosslinker content as determined by DSC (2nd heating run) and TGA.

Sample	DSC T_g_ (°C)	TGA T_5%_^a^ (°C)	TGA T_max_^b^ (°C)	TGA Residue at 800 °C (wt.%)
LiTFSI	-	369	413	7.8
GI-P(VImC_4_ TFSI)	19	357	416	5.1
GI-P(VImC_4_ TFSI-BAAP)_95:5_	32	344	406	4.0
GI-P(VImC_4_ TFSI-BAAP)_95:5_ + LiTFSI (10 mol%)	25	347	409	5.2
GII-P(AAC_6_ImC_1_ TFSI-BAAP)_95:5_	−29	368	405	7.1
GII-P(AAC_6_ImC_2_ TFSI-BAAP)_95:5_	−32	382	408	6.4
GII-P(AAC_6_ImC_4_ TFSI-BAAP)_95:5_	−41	363	400	5.8
GII-P(AAC_6_ImC_6_ TFSI-BAAP)_95:5_	−35	374	405	5.1
GII-P(AAC_9_ImC_4_ TFSI-BAAP)_95:5_	−40	376	405	5.5
GII-P(AAC_12_ImC_4_ TFSI-BAAP)_95:5_	−42	347	408	5.2
GII-P(AAC_6_ImC_1_ TFSI-BAAP)_95:5_ + LiTFSI (10 mol%)	−32	370	407	6.4
GII-P(AAC_6_ImC_2_ TFSI-BAAP)_95:5_ + LiTFSI (10 mol%)	−36	380	412	5.7
GII-P(AAC_6_ImC_4_ TFSI-BAAP)_95:5_ + LiTFSI (10 mol%)	−41	357	406	5.3
GII-P(AAC_6_ImC_6_ TFSI-BAAP)_95:5_ + LiTFSI (10 mol%)	−36	372	410	4.9
GII-P(AAC_9_ImC_4_ TFSI-BAAP)_95:5_ + LiTFSI (10 mol%)	−42	375	411	3.6
GII-P(AAC_12_ImC_4_ TFSI-BAAP)_95:5_ + LiTFSI (10 mol%)	−44	378	426	3.3
GIII-P(VImC_6_ImC_1_TFSI_2_-BAAP)_95:5_	−6	385	463	0.8
GIII-P(VImC_6_ImC_4_ TFSI_2_-BAAP)_95:5_	−13	398	463	6.3
GIII-P(VImC_12_ImC_1_TFSI_2_-BAAP)_95:5_	−24	384	448	3.4
GIII-P(VImC_12_ImC_4_TFSI_2_-BAAP)_95:5_	−31	385	446	4.4

^a^ T_5%_: temperature at which the polymer lost 5 wt.% of initial weight. Indicates decomposition start point. ^b^ T_max_: temperature at which the highest weight loss occurred.

**Table 2:** Calculation of network characteristics of [GII-P(AAC_6_ImC_4_ TFSI-BAAP)_x:y_] with different amounts of crosslinker and LiTFSI using the rheologically determined plateau modulus *G′* at 60 °C.

Entry	BAAP Content (mol%)	LiTFSI Content (mol%)	T_g,DSC_ (°C)	*G′*_60 °C_ (Pa)	*ν_eff_* (mol·m^3^)	L (nm)	T_g,tanδ_ (°C)
1 ^a)^	0	0	−44	5.60 × 10^4^	60.7	4.35	−23
2 ^a)^	0	10		4.20 × 10^4^	249.3	2.71	−21
3	5	0	−41	2.30 × 10^5^	249.3	2.71	−20
4	10	0	−39	1.01 × 10^6^	1095.0	1.66	−16
5	5	10	−41	2.00 × 10^5^	216.8	2.84	−20
6	10	10		4.20 × 10^5^	455.3	2.22	−19
7	5	20		1.90 × 10^5^	206.0	2.89	−20
8	5	30		2.09 × 10^5^	226.6	2.80	−21
9	5	10	−41	2.68 × 10^5^	290.5	2.58	−21

^a)^ Network only exists virtually (no crosslinker used); thus, the values have formal character.

**Table 3:** Calculation of the specific conductivity for DC plating-stripping experiments (30 °C) using current density (*I_density_*) and voltage values shown in Figure 16 and the cell geometry used (sample diameter: 5 mm; film thickness d: 121 μm) for PIL network GII-P(AAC_6_ImC_4_ TFSI-BAAP)_95:5 +_ 20 mol% LiTFSI.

Current Density	Specific Conductivity (2nd Cycle) (S·cm^−1^)	Specific Conductivity (10th Cycle) (S·cm^−1^)	Ionic Bulk Conductivity (Calculated from EIS, Figure 10) (S·cm^−1^)
1.27 μA cm^−2^	8.09 × 10^−8^	1.54 × 10^−7^	5.3 × 10^−6^
−1.27 μA cm^−2^	9.04 × 10^−7^	1.18 × 10^−6^	5.3 × 10^−6^
12.7 μA cm^−2^	6.15 × 10^−8^	6.68 × 10^−8^	5.3 × 10^−6^
−12.7 μA cm^−2^	5.30 × 10^−8^	5.12 × 10^−8^	5.3 × 10^−6^

## Supplementary Materials

The following are available online at https://www.mdpi.com/2073-4360/12/8/1707/s1. ^1^H NMR and ^13^C NMR data, DSC and TGA curves, rheology results, selected Nyquist plots from EIS, and an equivalent circuit for Relaxis 3 fitting.
