# Supplementary material for: In Situ Preparation of Crosslinked Polymer Electrolytes for Lithium Ion Batteries: A Comparison of Monomer Systems
Source: Polymers (Basel). 2020 Jul 30;12(8):1707. doi: 10.3390/polym12081707 (PMC7466031; doi:10.3390/polym12081707)
Supplement: Supplementary file 1 [file polymers-12-01707-s001.pdf]

IN SITU PREPARATION OF CROSSLINKED POLYMER ELECTROLYTES FOR LITHIUM ION BATTERIES: COMPARISON OF MONOMER SYSTEMS

Eike T. Röchow [1,2], Matthias Coeler [3], Doris Pospiech [1*], Oliver Kobsch [1], Elizaveta Mechtaeva [4], Roland Vogel [1], Brigitte Voit [1,2], Kristian Nikolowski [3], Mareike Wolter [3]

[1] Leibniz-Institut für Polymerforschung Dresden e.V., Hohe Str. 6, 01069 Dresden, Germany
[2] Technische Universität Dresden, Organic Chemistry of Polymers, 01062 Dresden, Germany
[3] Fraunhofer-Institut für Keramische Technologien und Systeme IKTS, Winterbergstr. 28, 01277 Dresden, Germany
[4] St. Petersburg University, Universitetskaya Emb., 7/9, Saint-Petersburg, 199034, Russia
* Correspondence: pospiech@ipfdd.de; Tel.: +49-351-4658-497

SUPPORTING INFORMATION

Supporting Information

Table of contents

1. NMR Data of Monomers 3

Generation I 3

Generation II 3

Generation III 4

2. DSC Data 5

3. TGA Data 7

4. Rheology Data 9

5. EIS Data 13

1. NMR Data of Monomers

Generation I

VImC$_4$ TFSI *^{1}H NMR (500 MHz, DMSO-d$_6$) δ_H ppm* 0.92 (t, *J*=7.41 Hz, 3 H) 1.19 - 1.39 (m, 2 H) 1.81 (quin, *J*=7.41 Hz, 2 H) 4.19 (t, *J*=7.25 Hz, 2 H) 5.42 (dd, *J*=8.51, 2.21 Hz, 1 H) 5.94 (dd, *J*=15.61, 2.36 Hz, 1 H) 7.27 (dd, *J*=15.76, 8.83 Hz, 1 H) 7.92 (t, *J*=1.70 Hz, 1 H) 8.18 (t, *J*=1.70 Hz, 1 H) 9.46 (s, 1 H); *^{13}C NMR (126 MHz, DMSO-d$_6$) δ_C ppm* 13.15 (s, 1 C) 18.74 (s, 1 C) 31.00 (s, 1 C) 48.95 (s, 1 C) 108.59 (s, 1 C) 119.46 (q, *J*=322.00 Hz, 1 C) 119.15 (s, 1 C) 122.29 - 125.36 (m, 1 C) 128.83 (s, 1 C) 135.26 (s, 1 C)

Generation II

AAC$_6$ImC$_1$ TFSI *^{1}H NMR (500 MHz, DMSO-d$_6$) δ_H ppm* 1.27 (quin, *J*=7.40 Hz, 2 H) 1.36 (quin, *J*=7.30 Hz, 2 H) 1.61 (quin, *J*=7.01 Hz, 2 H) 1.79 (quin, *J*=7.33 Hz, 2 H) 3.84 (s, 3 H) 4.10 (t, *J*=6.62 Hz, 2 H) 4.15 (t, *J*=7.09 Hz, 2 H) 5.93 (dd, *J*=10.40, 1.20 Hz, 1 H) 6.16 (dd, *J*=17.30, 10.20 Hz, 1 H) 6.31 (dd, *J*=17.40, 1.30 Hz, 1 H) 7.69 (s, 1 H) 7.75 (s, 1 H) 9.09 (s, 1 H); *^{13}C NMR (126 MHz, DMSO-d$_6$) δ_C ppm* 24.68 (s, 1 C) 25.03 (s, 1 C) 27.78 (s, 1 C) 29.16 (s, 1 C) 35.69 (s, 1 C) 48.65 (s, 1 C) 63.85 (s, 1 C) 119.44 (q, *J*=322.00 Hz, 2 C) 122.21 (s, 1 C) 123.56 (s, 1 C) 128.33 (s, 1 C) 131.28 (s, 1 C) 136.46 (s, 1 C) 165.46 (s, 1 C)

AAC$_6$ImC$_2$ TFSI *^{1}H NMR (500 MHz, DMSO-d$_6$) δ_H ppm* 1.28 (quin, *J*=7.30 Hz, 2 H) 1.36 (quin, *J*=7.30 Hz, 2 H) 1.43 (t, *J*=7.41 Hz, 3 H) 1.62 (quin, *J*=7.01 Hz, 2 H) 1.81 (quin, *J*=7.50 Hz, 2 H) 4.10 (t, *J*=6.62 Hz, 2 H) 4.15 (t, *J*=7.57 Hz, 2 H) 4.19 (q, *J*=7.30 Hz, 2 H) 5.93 (dd, *J*=10.25, 1.42 Hz, 1 H) 6.16 (dd, *J*=17.30, 10.20 Hz, 1 H) 6.31 (dd, *J*=17.18, 1.42 Hz, 1 H) 7.79 (d, *J*=8.20 Hz, 2 H) 9.17 (s, 1 H); *^{13}C NMR (126 MHz, DMSO-d$_6$) δ_C ppm* 14.90 (s, 2 C) 24.67 (s, 1 C) 24.85 - 25.56 (m, 1 C) 27.77 (s, 1 C) 29.11 (s, 1 C) 44.18 (s, 1 C) 48.73 (s, 1 C) 63.83 (s, 1 C) 119.45 (q, *J*=322.00 Hz, 2 C) 122.08 (s, 1 C) 122.35 (s, 1 C) 128.33 (s, 1 C) 131.26 (s, 1 C) 135.60 (s, 1 C) 165.45 (s, 1 C)

AAC$_6$ImC$_4$ TFSI *^{1}H NMR (500 MHz, DMSO-d$_6$) δ_H ppm* 0.90 (t, *J*=7.25 Hz, 3 H) 1.26 (quin, *J*=7.41 Hz, 4 H) 1.35 (quin, *J*=7.50 Hz, 2 H) 1.61 (quin, *J*=7.09 Hz, 2 H) 1.79 (dquin, *J*=14.70, 7.30 Hz, 4 H) 4.09 (t, *J*=6.62 Hz, 2 H) 4.15 (td, *J*=7.09, 1.89 Hz, 4 H) 5.93 (dd, *J*=10.40, 1.58 Hz, 1 H) 6.16 (dd, *J*=17.30, 10.20 Hz, 1 H) 6.31 (dd, *J*=17.18, 1.42 Hz, 1 H) 7.78 (d, *J*=1.26 Hz, 2 H) 9.17 (s, 1 H); *^{13}C NMR (126 MHz, DMSO-d$_6$) δ_C ppm* 13.16 (s, 1 C) 18.72 (s, 1 C) 24.64 (s, 1 C) 25.03 (s, 1 C) 27.78 (s, 1 C) 29.05 (s, 1 C) 30.12 - 33.68 (m, 1 C) 48.56 (s, 1 C) 48.74 (s, 1 C) 63.82 (s, 1 C) 119.44 (q, *J*=322.00 Hz, 2 C) 122.41 (s, 2 C) 128.32 (s, 1 C) 131.29 (s, 1 C) 135.88 (s, 1 C) 165.45 (s, 1 C)

AAC$_6$ImC$_6$ TFSI *^{1}H NMR (500 MHz, DMSO-d$_6$) δ_H ppm* 0.85 (t, *J*=7.20 Hz, 3 H) 1.17 - 1.31 (m, 8 H) 1.36 (quin, *J*=7.50 Hz, 2 H) 1.61 (quin, *J*=7.50 Hz, 2 H) 1.80 (sxt, *J*=7.50 Hz, 4 H) 4.09 (t, *J*=6.46 Hz, 2 H) 4.16 (td, *J*=7.17, 3.00 Hz, 4 H) 5.93 (dd, *J*=10.25, 1.42 Hz, 1 H) 6.16 (dd, *J*=17.30, 10.20 Hz, 1 H) 6.31 (dd, *J*=17.18, 1.42 Hz, 1 H) 7.78 (d, *J*=1.58 Hz, 2 H) 9.18 (s, 1 H); *^{13}C NMR (126 MHz, DMSO-d$_6$) δ_C ppm* 13.67 (s, 1 C) 21.78 (s, 1 C) 24.64 (s, 1 C) 25.03 (s, 1 C) 25.06 (s, 1 C) 27.79 (s, 1 C) 29.06 (s, 1 C) 29.15 (s, 1 C) 30.42 (s, 1 C) 48.75 (s, 1 C)

48.83 (s, 1 C) 63.81 (s, 1 C) 119.45 (q, *J*=322.00 Hz, 2 C) 122.41 (s, 2 C) 128.32 (s, 1 C) 131.22 (s, 1 C) 135.88 (s, 1 C) 165.43 (s, 1 C)

AAC_9ImC_4 TFSI *^1H NMR (500 MHz, DMSO-d_6) δ_H ppm* 0.90 (t, *J*=7.41 Hz, 3 H) 1.11 - 1.40 (m, 12 H) 1.60 (quin, *J*=6.86 Hz, 2 H) 1.79 (sxt, *J*=7.30 Hz, 4 H) 4.09 (t, *J*=6.62 Hz, 2 H) 4.16 (q, *J*=6.52 Hz, 4 H) 5.92 (dd, *J*=10.40, 1.58 Hz, 1 H) 6.15 (dd, *J*=17.30, 10.20 Hz, 1 H) 6.31 (dd, *J*=17.40, 1.60 Hz, 1 H) 7.78 (d, *J*=1.58 Hz, 2 H) 9.18 (s, 1 H); *^{13}C NMR (126 MHz, DMSO-d_6) δ_C ppm* 13.10 (s, 1 C) 18.70 (s, 1 C) 25.23 (s, 1 C) 25.37 (s, 1 C) 28.00 (s, 1 C) 28.13 (s, 1 C) 28.39 (s, 1 C) 28.58 (s, 1 C) 29.16 (s, 1 C) 31.20 (s, 1 C) 48.56 (s, 1 C) 48.83 (s, 1 C) 63.96 (s, 1 C) 199.45 (q, *J*=322.00 Hz, 2 C) 122.39 (s, 2 C) 128.36 (s, 1 C) 131.10 (s, 1 C) 135.86 (s, 1 C) 165.44 (s, 1 C)

$AAC_{12}ImC_4$ TFSI *^1H NMR (500 MHz, DMSO-d_6) δ_H ppm* 0.90 (t, *J*=7.10 Hz, 3 H) 1.16 - 1.34 (m, 18 H) 1.60 (quin, *J*=6.86 Hz, 2 H) 1.77 (quin, *J*=7.30 Hz, 4 H) 4.09 (t, *J*=6.62 Hz, 2 H) 4.15 (q, *J*=6.94 Hz, 4 H) 5.93 (dd, *J*=10.25, 1.42 Hz, 1 H) 6.16 (dd, *J*=17.30, 10.20 Hz, 1 H) 6.31 (dd, *J*=17.40, 1.60 Hz, 1 H) 7.78 (d, *J*=1.26 Hz, 2 H) 9.18 (s, 1 H); *^{13}C NMR (126 MHz, DMSO-d_6) δ_C ppm* 13.14 (s, 1 C) 18.71 (s, 1 C) 25.27 (s, 1 C) 25.40 (s, 1 C) 28.01 (s, 1 C) 28.24 (s, 1 C) 28.53 (s, 1 C) 28.72 (s, 1 C) 28.78 (s, 1 C) 28.82 (s, 3 C) 29.18 (s, 1 C) 31.21 (s, 1 C) 48.56 (s, 1 C) 48.83 (s, 1 C) 63.99 (s, 1 C) 119.45 (q, *J*=322.00 Hz, 2 C) 122.41 (s, 1 C) 128.37 (s, 1 C) 131.17 (s, 1 C) 135.87 (s, 1 C) 165.46 (s, 1 C)

Generation III

VImC6ImC4 2TFSI *^1H NMR (500 MHz, DMSO-d_6) δ_H ppm* 0.90 (t, *J*=7.41 Hz, 3 H) 1.12 - 1.45 (m, 6 H) 1.63 - 2.03 (m, 6 H) 3.96 - 4.25 (m, 6 H) 5.43 (dd, *J*=8.67, 2.36 Hz, 1 H) 5.94 (dd, *J*=15.61, 2.36 Hz, 1 H) 7.28 (dd, *J*=15.61, 8.67 Hz, 1 H) 7.78 (d, *J*=11.03 Hz, 2 H) 7.90 (s, 1 H) 8.18 (s, 1 H) 9.16 (s, 1 H) 9.45 (s, 1 H); *^{13}C NMR (126 MHz, DMSO-d_6) δ_C ppm* 13.16 (s, 1 C) 18.72 (s, 1 C) 24.87 (s, 1 C) 24.92 (s, 1 C) 28.79 (s, 1 C) 29.00 (s, 1 C) 31.22 (s, 1 C) 48.57 (s, 1 C) 48.72 (s, 1 C) 49.08 (s, 1 C) 108.65 (s, 1 C) 119.14 (s, 1 C) 119.44 (q, *J*=322.00 Hz, 4 C) 122.39 (s, 1 C) 122.43 (s, 1 C) 123.17 (s, 1 C) 128.82 (s, 1 C) 135.24 (s, 1 C) 135.84 (s, 1 C)

VImC12ImC4 2TFSI *^1H NMR (500 MHz, DMSO-d_6) δ_H ppm* 0.90 (t, *J*=7.41 Hz, 3 H) 1.13 - 1.35 (m, 18 H) 1.69 - 1.89 (m, 6 H) 4.16 (quin, *J*=7.65 Hz, 6 H) 5.42 (dd, *J*=8.67, 2.36 Hz, 1 H) 5.94 (dd, *J*=15.61, 2.36 Hz, 1 H) 7.27 (dd, *J*=15.61, 8.67 Hz, 1 H) 7.78 (s, 2 H) 7.90 (s, 1 H) 8.17 (s, 1 H) 9.17 (s, 1 H) 9.45 (s, 1 H); *^{13}C NMR (126 MHz, DMSO-d_6) δ_C ppm* 13.16 (s, 1 C) 18.71 (s, 1 C) 25.47 (s, 2 C) 28.32 (s, 1 C) 28.37 (s, 1 C) 28.75 - 28.95 (m, 4 C) 29.02 (s, 1 C) 29.21 (s, 1 C) 31.20 (s, 1 C) 48.55 (s, 1 C) 48.83 (s, 1 C) 49.20 (s, 1 C) 108.60 (s, 1 C) 119.44 (q, *J*=322.00 Hz, 4 C) 119.11 (s, 1 C) 122.41 (s, 2 C) 123.19 (s, 1 C) 128.84 (s, 1 C) 135.23 (s, 1 C) 135.86 (s, 1 C)

2. DSC Data

DSC curves (2nd heating curves to eliminate influences of thermal history) are shown which were used to determine T_g values for PIL networks with and without additional LiTFSI.

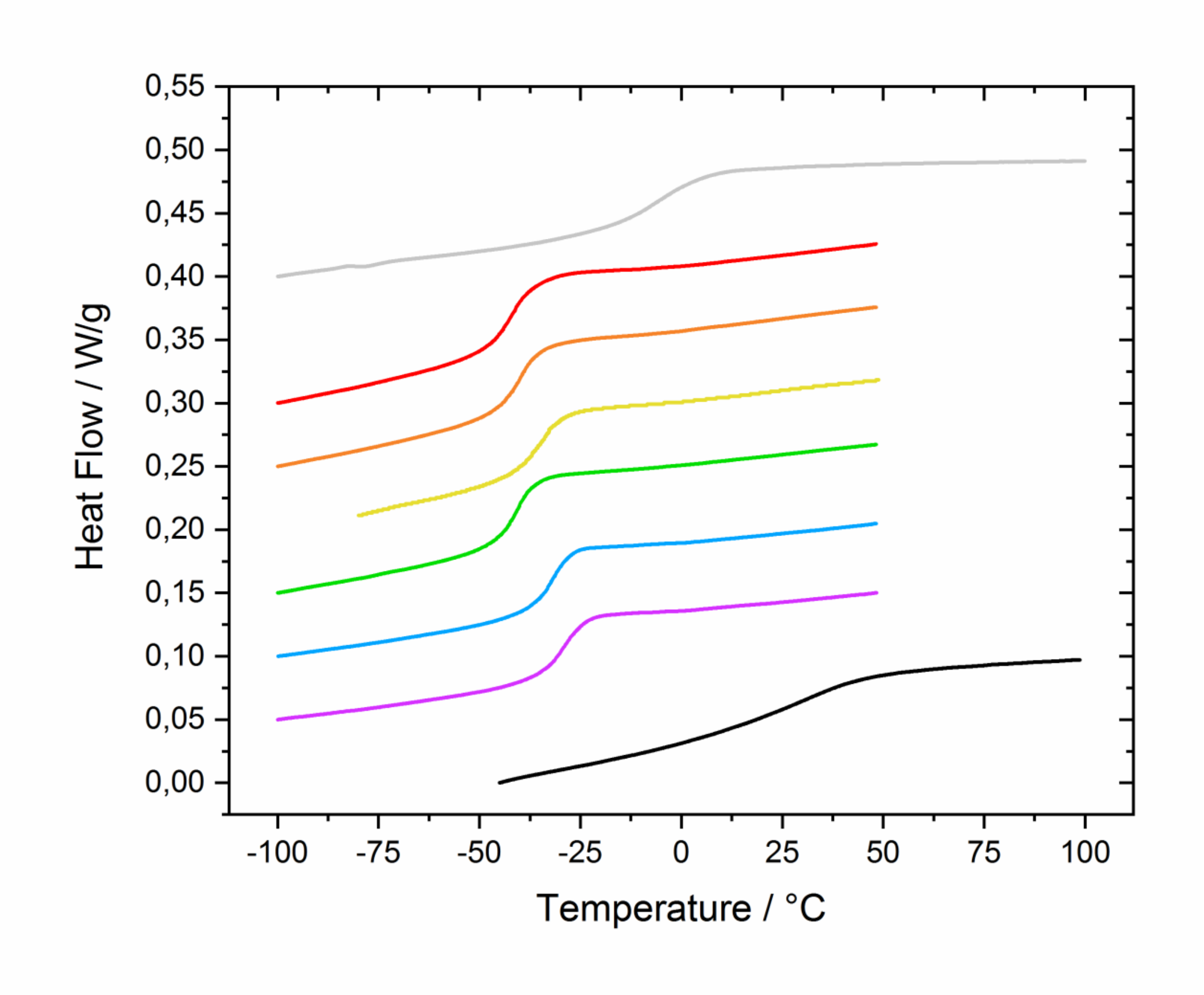

Figure SI.1: DSC curves of PIL networks:

(black) GI-P(VImC$_4$ TFSI-BAAP)$_{95:5}$, (violet) GII-P(AAC$_6$ImC$_1$ TFSI-BAAP)$_{95:5}$,

(blue) GII-P(AAC$_6$ImC$_2$ TFSI-BAAP)$_{95:5}$, (green) GII-P(AAC$_6$ImC$_4$ TFSI-BAAP)$_{95:5}$,

(yellow) GII-P(AAC$_6$ImC$_6$ TFSI-BAAP)$_{95:5}$, (orange) GII-P(AAC$_9$ImC$_4$ TFSI-BAAP)$_{95:5}$,

(red) GII-P(AAC$_{12}$ImC$_4$ TFSI-BAAP)$_{95:5}$, (grey) GIII-P(VImC$_6$ImC$_4$ TFSI-BAAP)$_{95:5}$.

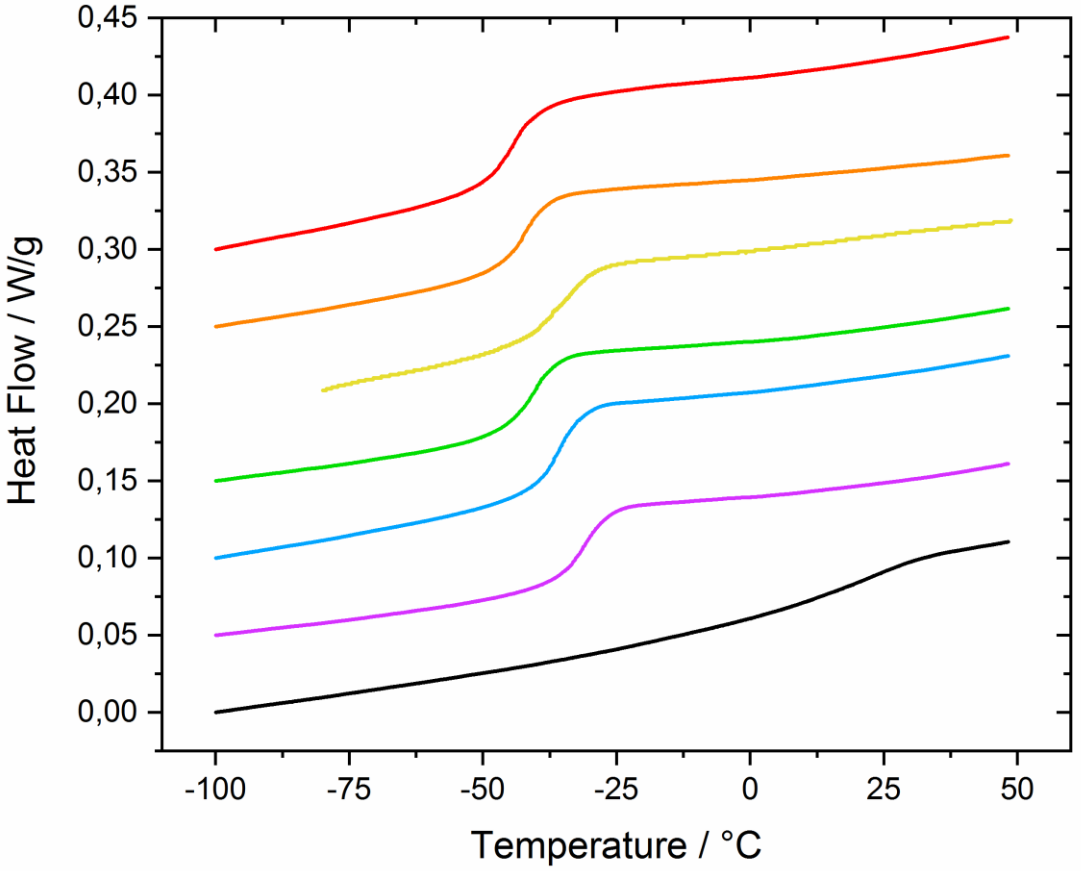

Figure SI.2: DSC curves of PIL networks with additional amount of 10 mol% LiTFSI: (black) GI-P($VImC_4$ TFSI-BAAP)$_{95:5}$, (violet) GII-P(AAC_6ImC_1 TFSI-BAAP)$_{95:5}$, (blue) GII-P(AAC_6ImC_2 TFSI-BAAP)$_{95:5}$, (green) GII-P(AAC_6ImC_4 TFSI-BAAP)$_{95:5}$, (yellow) GII-P(AAC_6ImC_6 TFSI-BAAP)$_{95:5}$, (orange) GII-P(AAC_9ImC_4 TFSI-BAAP)$_{95:5}$, (red) GII-P($AAC_{12}ImC_4$ TFSI-BAAP)$_{95:5}$.

3. TGA Data

The TGA curves shown in Figure SI.3 and Figure SI.4 were used to derive the parameters of thermal decomposition of PIL networks as given in Table 1.

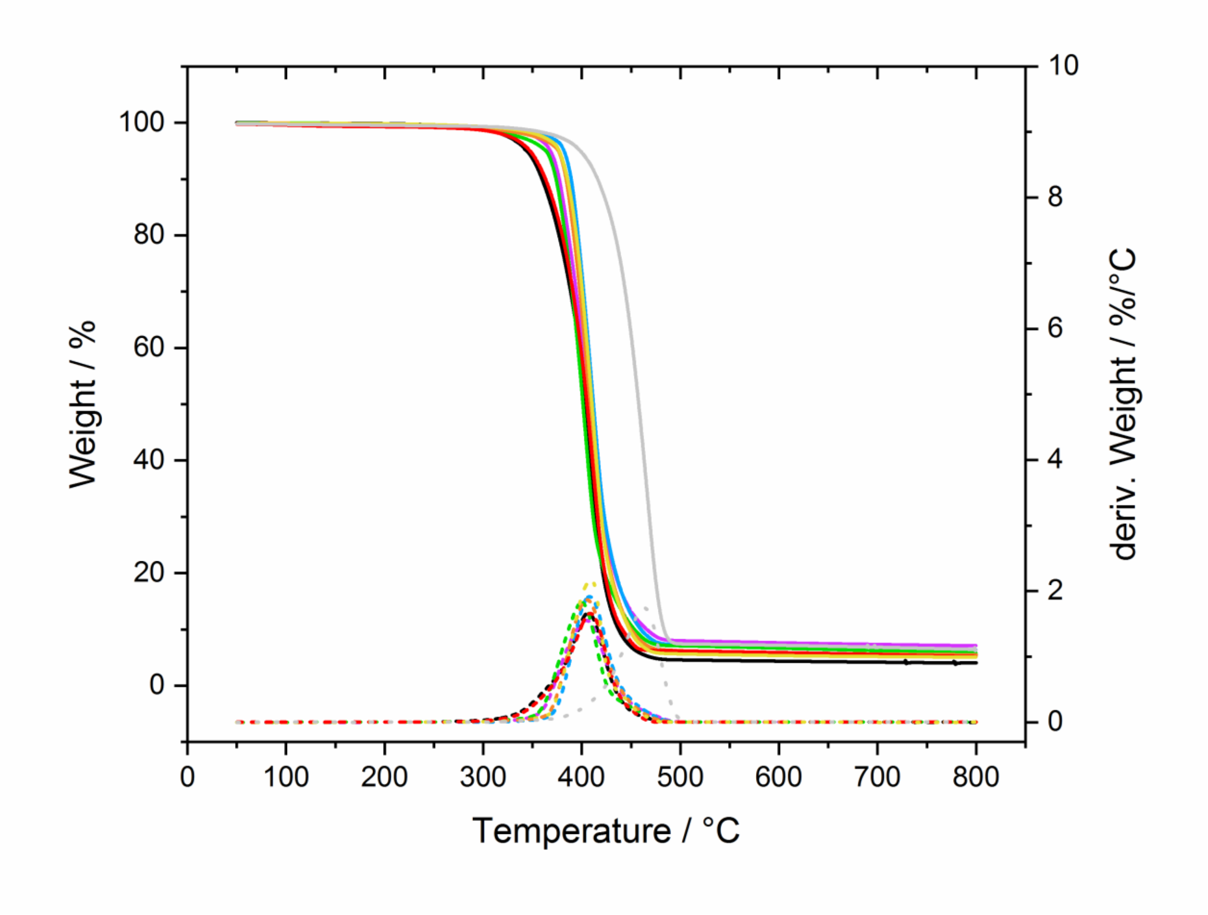

Figure SI.3: TGA curves of PIL networks without additional LiTFSI: (black) GI-P(VImC$_4$ TFSI-BAAP)$_{95:5}$, (violet) GII-P(AAC$_6$ImC$_1$ TFSI-BAAP)$_{95:5}$, (blue) GII-P(AAC$_6$ImC$_2$ TFSI-BAAP)$_{95:5}$, (green) GII-P(AAC$_6$ImC$_4$ TFSI-BAAP)$_{95:5}$, (yellow) GII-P(AAC$_6$ImC$_6$ TFSI-BAAP)$_{95:5}$, (orange) GII-P(AAC$_9$ImC$_4$ TFSI-BAAP)$_{95:5}$, (red) GII-P(AAC$_{12}$ImC$_4$ TFSI-BAAP)$_{95:5}$, (grey) GIII-P(VImC$_6$ImC$_4$ TFSI-BAAP)$_{95:5}$.

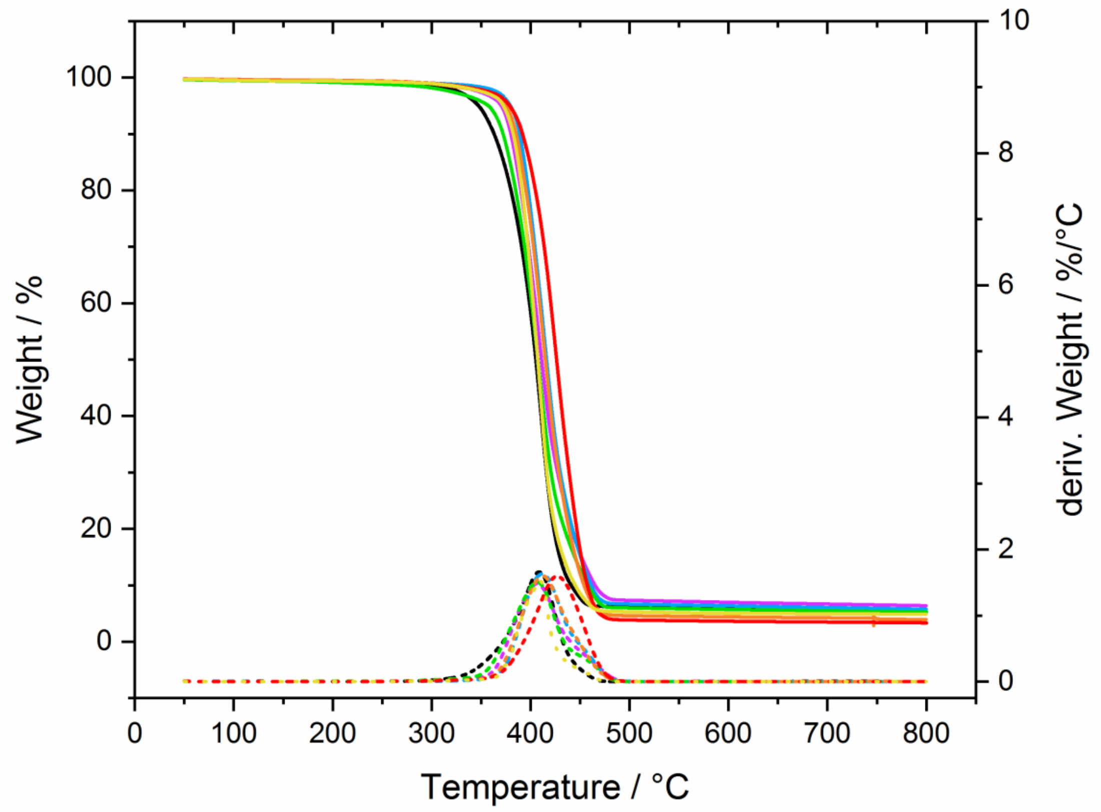

Figure SI.4: TGA curves of PIL networks with additional amount of 10 mol% LiTFSI: (black) GI-P(VImC$_4$ TFSI-BAAP)$_{95:5}$, (violet) GII-P(AAC$_6$ImC$_1$ TFSI-BAAP)$_{95:5}$, (blue) GII-P(AAC$_6$ImC$_2$ TFSI-BAAP)$_{95:5}$, (green) GII-P(AAC$_6$ImC$_4$ TFSI-BAAP)$_{95:5}$, (yellow) GII-P(AAC$_6$ImC$_6$ TFSI-BAAP)$_{95:5}$, (orange) GII-P(AAC$_9$ImC$_4$ TFSI-BAAP)$_{95:5}$, (red) GII-P(AAC$_{12}$ImC$_4$ TFSI-BAAP)$_{95:5}$.

4. Rheology Data

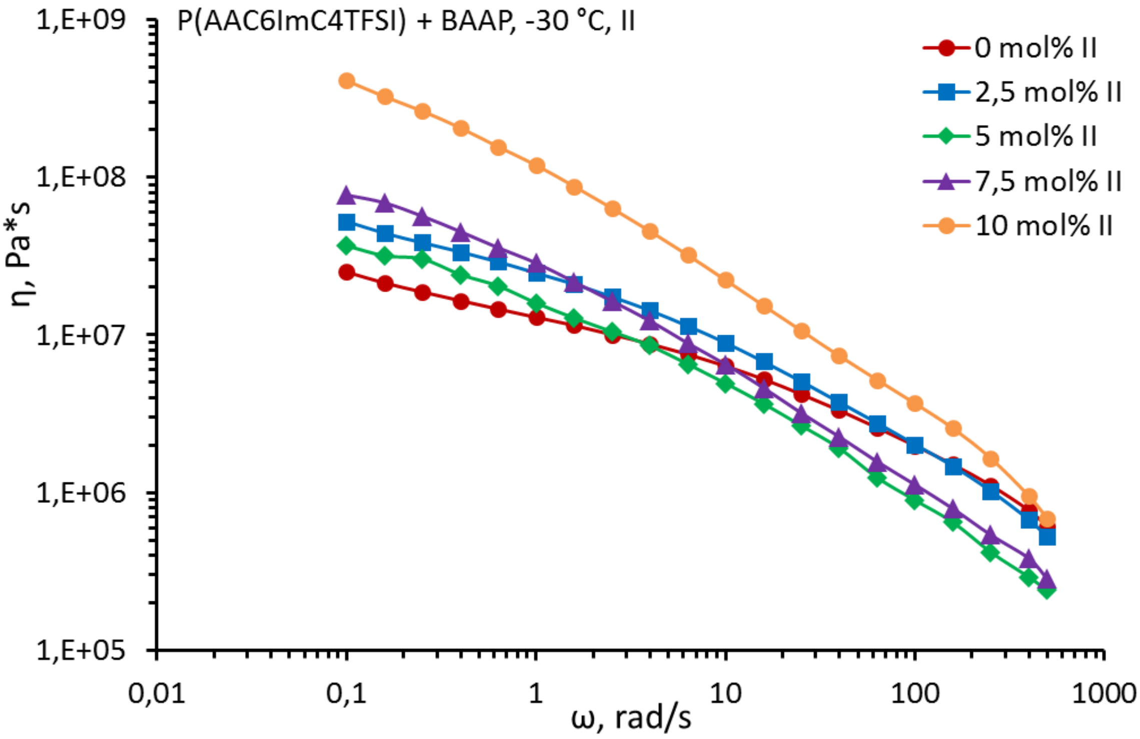

Figure SI.5: Dependence of the apparent viscosity on the angular frequency for the polymer networks GII-P(AAC_6ImC_4 TFSI-BAAP)$_{x:y}$ with different amounts of the crosslinker BAAP at T = –30 °C.

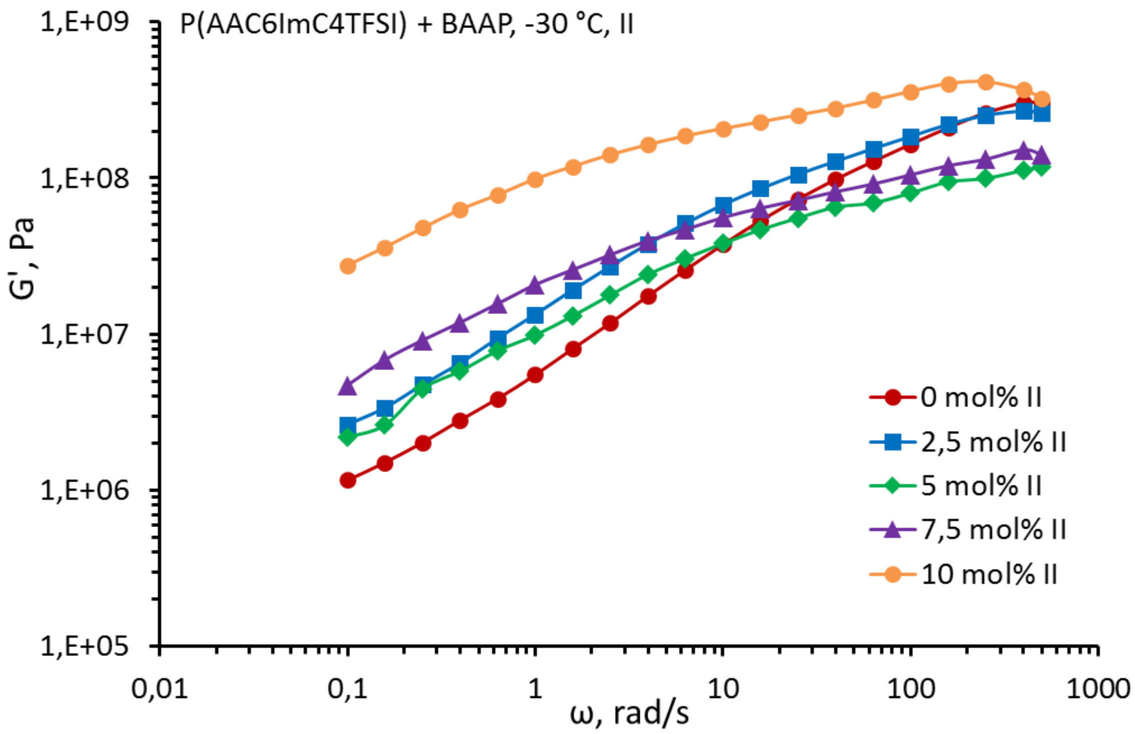

Figure SI.6: Dependence of the storage modulus on the angular frequency for the polymer networks GII-P(AAC_6ImC_4 TFSI-BAAP)$_{x:y}$ amounts of the crosslinker BAAP at T = –30 °C.

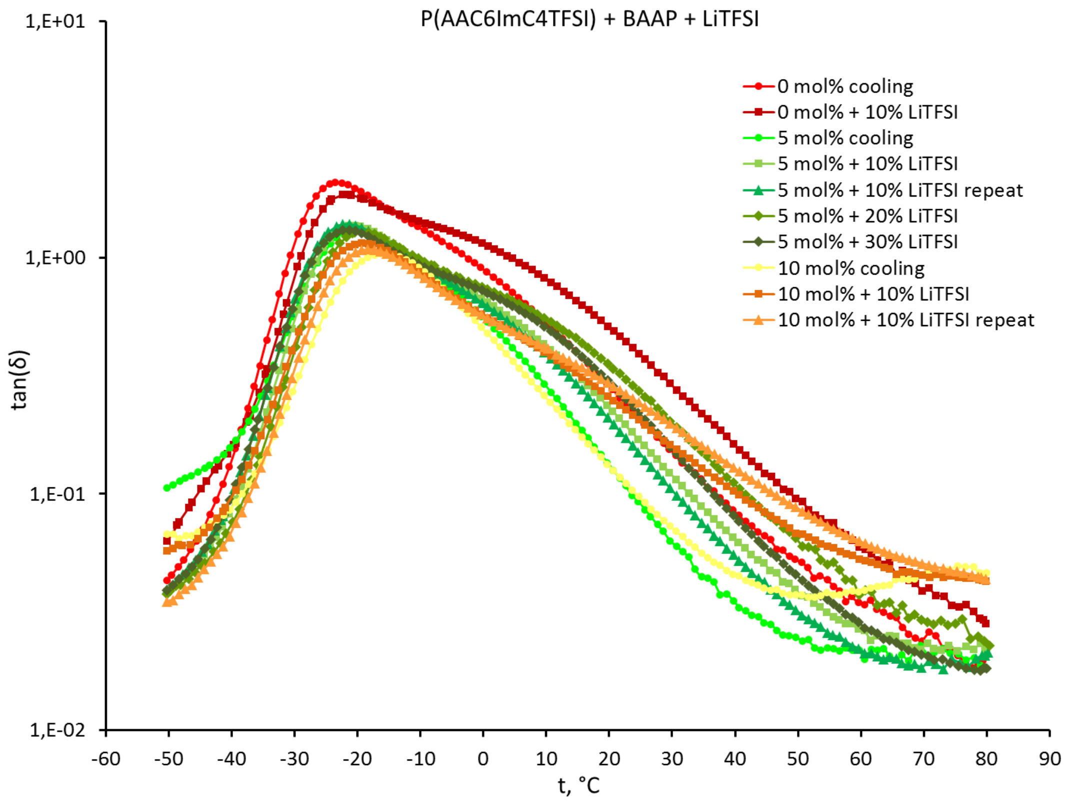

Figure SI.7: Dependence of the tan(δ) on the temperature for the polymer P(AAC_6ImC_4 TFSI-BAAP) with different amounts of the crosslinker BAAP and different amounts of LiTFSI salt.

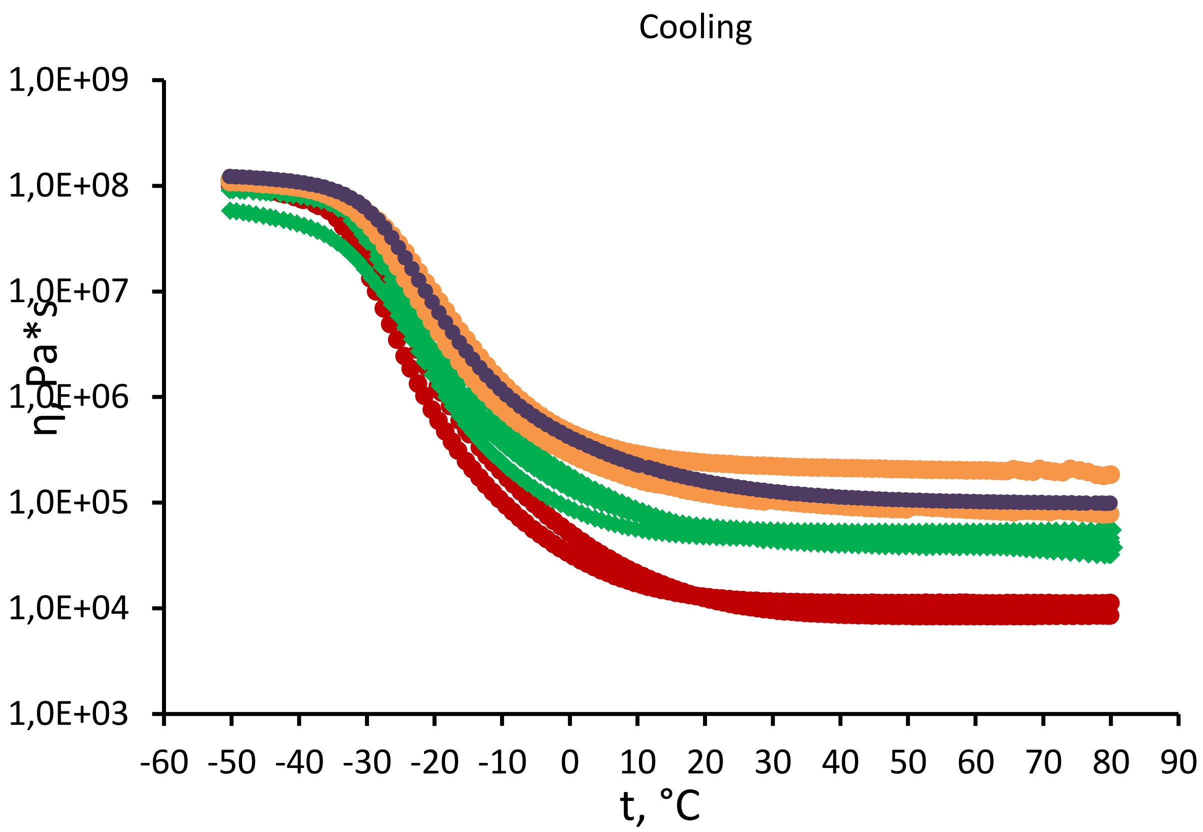

Figure SI.8: Dependence of the complex viscosity η on the temperature for the polymer P(AAC_6ImC_4 TFSI-BAAP) with different amounts of the crosslinker BAAP and different amounts of LiTFSI salt.

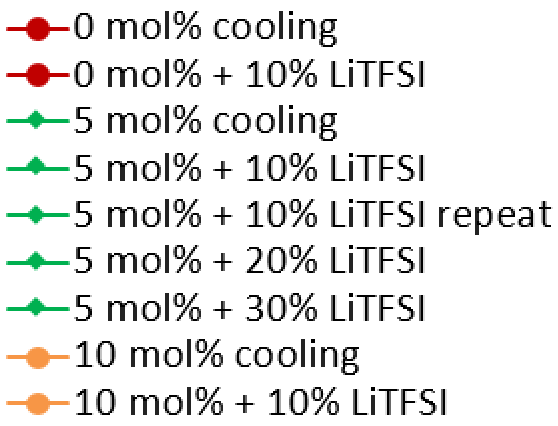

5. EIS Data

As an example of EIS, results obtained for the sample P(VImC$_4$ TFSI-BAAP)$_{95:5}$ vs Li/Li$^+$ are shown in a Nyquist plot. There, two semicircles can be seen. The first semicircle corresponds to bulk resistance and is used for calculation of ionic conductivity. The second semicircle stands for interface resistance and reflects the transition of lithium ions between polymer electrolyte and the lithium electrode. It can be noted that with increasing temperature resistances are decreasing.

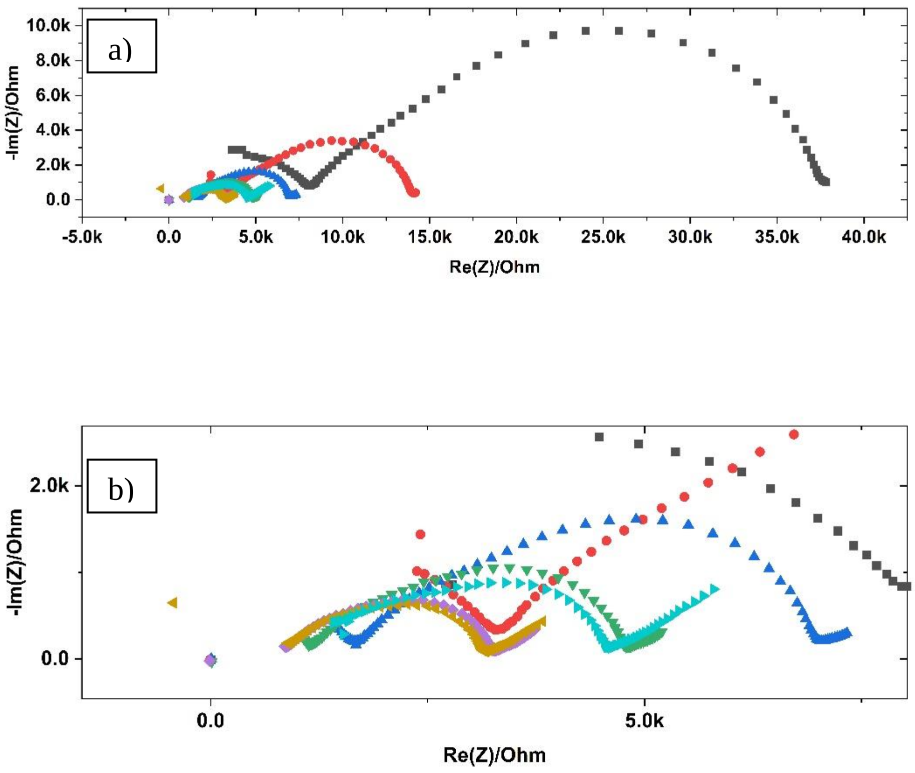

Figure SI.9: a) Example of typical Nyquist plots of GI-P(VImC$_4$ TFSI-BAAP)$_{95:5}$; **b)** zoomed in; black: 20 °C, red: 30 °C, blue: 40 °C, green; 50 °C, violet: 60 °C, yellow: 70 °C, teal: 80 °C.

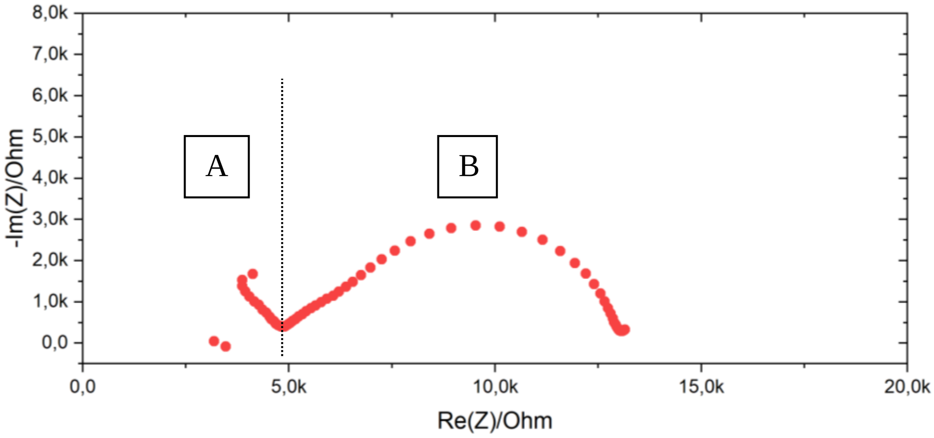

Figure SI.10: Nyquist plot of sample GII-P(AAC_6ImC_4 TFSI-BAAP)$_{95:5}$ + 20 mol% LiTFSI at 30 °C; A: Bulk resistance R_b; Transfer Resistance defined by semicircle B: 9878 Ω with Relaxis3 Software using equivalent circuit of SI.11.

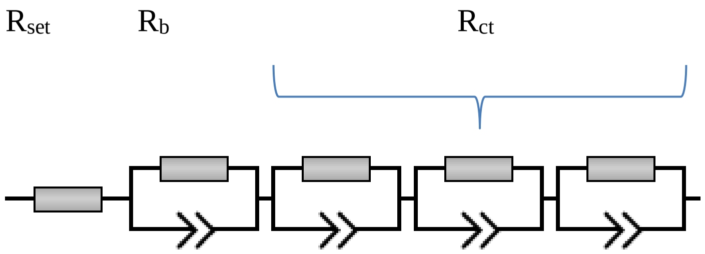

Figure SI.11: Equivalent circuit used as fitting model for EIS measurements by Relaxis3 software, obtained by the model screening algorithm. The first resistance R_{set} describes the electrical loss of the cell setup, the second resistance capacitor (RC) (R_b) describes the first semicircle, which determines the bulk conductivity. The following three RCs describe charge transfer processes (R_{ct}), assumed to have three overlaying processes and resulting in a not perfectly shaped semicircle.

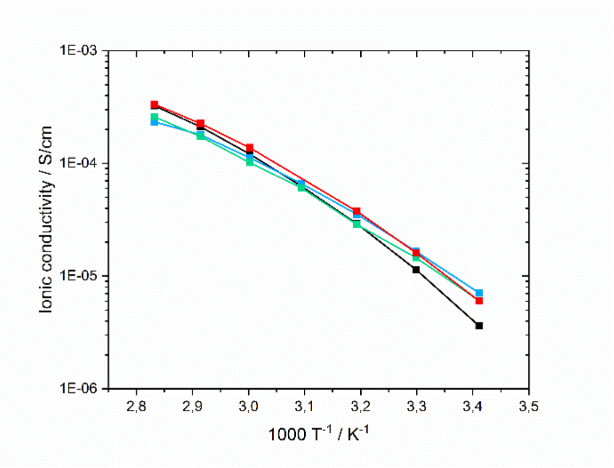

Figure SI.12: Influence of the N-alkyl substituent length on the ionic conductivity of (GII P(AAC6ImCx TFSI BAAP)95:5) with x = 1; 2; 4; 6) networks in the temperature range between 20 and 80 °C: (black) GII-P(AAC6ImC1 TFSI-BAAP)95:5; (red) GII-P(AAC6ImC2 TFSI-BAAP)95:5; (blue) GII-P(AAC6ImC4 TFSI-BAAP)95:5; GII-P(AAC6ImC6 TFSI-BAAP)95:5.
